# High Copy-Number Variation Burdens in Cranial Meningiomas From Patients With Diverse Clinical Phenotypes Characterized by Hot Genomic Structure Changes

**DOI:** 10.3389/fonc.2020.01382

**Published:** 2020-08-14

**Authors:** Junpeng Ma, Yaqiang Hong, Wei Chen, Da Li, Kaibing Tian, Ke Wang, Yang Yang, Yuan Zhang, Yujia Chen, Lairong Song, Liangpeng Chen, Liwei Zhang, Jiang Du, Junting Zhang, Zhen Wu, Dake Zhang, Liang Wang

**Affiliations:** ^1^Department of Neurosurgery, Beijing Tiantan Hospital, Capital Medical University, Beijing, China; ^2^Tsinghua-Peking Center for Life Sciences, School of Life Sciences, Tsinghua University, Beijing, China; ^3^Beijing Advanced Innovation Center for Biomedical Engineering, School of Biological Science and Medical Engineering, Beihang University, Beijing, China; ^4^Key Laboratory of Genomic and Precision Medicine, Beijing Institute of Genomics, Chinese Academy of Sciences, Beijing, China; ^5^China National Clinical Research Center for Neurological Diseases, Beijing, China; ^6^Center of Brain Tumor, Beijing Institute for Brain Disorders, Beijing, China; ^7^Beijing Key Laboratory of Brain Tumor, Beijing, China; ^8^Department of Neuropathology, Beijing Neurological Institute, Capital Medical University, Beijing, China

**Keywords:** copy number variation, female prominence, multiple meningiomas, oncogenic driver, recurrence, tumor location

## Abstract

Meningiomas, as the most common primary tumor of the central nervous system, are known to harbor genomic aberrations that associate with clinical phenotypes. Here we performed genome-wide genotyping for cranial meningiomas in 383 Chinese patients and identified 9,821 copy-number variations (CNVs). Particularly, patients with diverse clinical features had distinct tumor CNV profiles. CNV burdens were greater in high-grade (WHO grade II and III) samples, recurrent lesions, large tumors (diameter >4.3 cm), and those collected from male patients. Nevertheless, the level of CNV burden did not relate to tumor locations, peritumoral brain edema, bone invasion, or multiple lesions. Overall, the most common tumor CNVs were the copy-number gain (CNG) at 22q11.1 and the copy-number losses (CNLs) at 22q13.2, 14q11.2, 1p34.3, and 1p31.3. Recurrent lesions were featured by the CNLs at 1p31.3, 6q22.31, 9p21.3, and 11p12, and high-grade samples had more CNVs at 4q13.3 and 6q22.31. Meanwhile, large tumors were more likely to have the CNVs at 1p31.3 and 1p34.3. Additionally, recurrence prediction indicated the CNLs at 4p16.3 (*p* = 0.009, hazard ratio = 5.69) and 10p11.22 (*p* = 0.037, hazard ratio = 4.53) were candidate independent risk factors.

## Introduction

Meningiomas represent the most common primary intracranial tumor type, accounting for 37% of central nervous system neoplasms (1). They are believed to arise from progenitor cells of both the arachnoid cap cells of the arachnoid layer and fibroblasts that reside in the inner dura mater (2). Despite the identification of *NF2* mutations or loss of function, recent sequencing studies also revealed mutations involving *TRAF7, KLF4, AKT1, SMO, POLR2A*, and the *ARID1A* and *TERT* promoters of in meningiomas (3–6). Cytogenetic changes, such as losses of chromosomes 22q, 14q, 1p, 1q, 10q, and 9q, are also commonly reported, some of which are related to tumor progression (7–12). Copy-number variations (CNVs) of cytobands located at 22q, 1p, and 14q were most common (12–14). In tumor development, losses of 6q and 4q have been reported to be significantly associated with high-grade lesions (13). Furthermore, meningiomas in specific locations may have featured CNVs; for instance, those at anterior skull base are likely to have intact chromosome 22q, which loses tumor suppressor gene *NF2* (15). However, as a relative benign tumor, meningioma had few data from a relatively large cohort to characterize genome-wide CNV changes, which limits efforts on applying them in tumor progression evaluation, prognosis, and the development of new treatments.

Although maximal but safe resection can cure the majority of meningiomas (16), tumor recurrence still occurs even after gross total resection (GTR) (17). The recurrence status cannot be completely predicted by histopathologic grade alone, as it is mainly based on histopathological characterizations of mitotic rate, cellular features of atypia, and local invasion (18). Meningiomas are well-known for their female-biased predominance (19), but tumors in male patients demonstrate not only a higher annual growth rate (20) but also a higher probability of recurrence (21–23). Previous studies have proposed molecular markers for prognostic scoring systems in recent years (14, 21, 24–26), and a better WHO classification of meningiomas integrated with independent molecular markers may help to predict the recurrence risk and adjust treatment plans for patients with meningiomas. Although genomic structure changes in neurologic tumors are common, extensive efforts are still required to evaluate roles of diverse recurrent CNVs in the models for tumor classification, prognosis scoring, and recurrence prediction.

To our knowledge, we here collected cranial meningiomas by far at the largest sample size in the Chinese population. We performed genome-wide genotyping for all these samples and identified diverse common CNVs. Along with detailed clinical information, we investigated their relations with gender difference, tumor location, grade classification, and recurrence, and we further proposed candidate predictors for tumor recurrence.

## Materials and Methods

### Sample Collection

This study was approved by the Institutional Review Board of Beijing Tiantan Hospital affiliated with Capital Medical University. Three hundred and eighty-three frozen meningioma samples were collected at Beijing Tiantan Hospital, Capital Medical University, between August 2008 and August 2017. Signed informed consent forms were acquired from all patients or their guardians before surgery. Tumor specimens from meningioma samples were stored in liquid nitrogen immediately following collection. Genomic DNA was purified from tumor samples using a Biomek 3000 automated workstation with an E.Z.N.A Mag-Bind Tissue DNA Kit (Omega Bio-Tek, Norcross, GA, USA). DNA quality and quantity were determined using a NanoDrop 1000 instrument (Thermo Scientific, Wilmington, DE, USA).

### Clinical Data Collection and Follow-Up

Clinical information for 383 patients, including gender, age, primary or recurrent, degree of resection, tumor location, tumor diameter, bone invasion, peritumoral brain edema, pathological subtype, WHO grade, and follow-up results (recurrence and survival), was collected and summarized in Table 1. Pathological diagnosis was reviewed according to the 2016 WHO classification for meningiomas. Tumor recurrence was defined as tumor reemergence after GTR (gross total resection), or tumor regrowth with a minimum change of 25% increase of any tumor diameter after non-GTR based on contrast-enhanced MRIs (27). The degree of resection was decided according to the criteria of Simpson grading and classified as GTR (Simpson grade I to III) or STR (subtotal resection), verified by postoperative magnetic resonance images (MRIs) (28). Recurrence-free survival was defined as the period from the time of present surgery in our hospital to tumor recurrence (or last follow-up visit).

**Table 1:** The clinicopathological features of the entire cohort and subcohort in the prognostic analysis for recurrence.

		**Entire cohort**	**Subcohort**
Gender	Female	265	198
	Male	118	69
History of surgery	Primary	338	267
	Recurrent	45	0
WHO grade	I	331	241
	II	46	26
	III	6	0
Pathological subtype	Anaplastic	6	0
	Angiomatous	14	11
	Atypical	18	8
	Chondroid	5	3
	Clear cell	5	2
	Fibrous	66	58
	Lymphoplasmacyte rich	1	0
	Meningothelial	117	74
	Metaplastic	1	1
	Transitional	140	100
	Microcystic	4	4
	Psammomatous	2	2
	Secretory	4	4
Tumor location	Skull base	239	148
	Non-skull base	144	119
Bone invasion	Yes	57	38
	No	326	229
Peritumoral brain edema	Yes	118	84
	No	265	183
Multiple meningiomas	Yes	24	16
	No	359	251
Tumor size	> 4.3 cm	192	117
	≤ 4.3 cm	191	150
Degree of resection	Simpson I	122	109
	Simpson II	132	120
	Simpson III	48	38
	Simpson IV	81	0

### Whole-Genome Single-Nucleotide Polymorphism (SNP) Genotyping and Statistical Analysis

Whole-genome SNP array analysis for 383 meningioma samples was performed on Illumina Human Infinium CoreExome BeadChips (Illumina, San Diego, CA, USA). Raw intensity values were processed to obtain a normalized B allele frequency (BAF) and a log R ratio (LRR) for each probe using the GenomeStudio Software v2.0.4 (Illumina, San Diego, USA). LRR values were segmented with Genome Alteration Detection Analysis (GADA, Juan R. González, Barcelona, Spain) using parameters of *T* > 10 and segment lengths containing ≥50 continuous probes. For loss of heterozygosity (LOH) analysis, the sliding window approach was used with a window size of 100 informative SNPs. A window was considered to represent LOH if more than 80% of the SNPs had a minor allele frequency ≤0.9. A segment was defined either as normal or as having one of 3 types of alteration status based on the following criteria: (1) normal, |LRR| < 0.075 and retaining heterozygosity; (2) gain, LRR ≥ 0.075; (3) loss, LRR ≤ −0.075; and (4) copy-number neutral loss of heterozygosity (CNNLOH), |LRR| < 0.075.

To assess genome instability, the genomic fractions of CNVs, and CNNLOH were estimated by dividing the number of SNPs undergoing a specific alteration by the total number of SNPs present in the respective chromosome or in the respective sample. To identify minimal common regions (MCRs) of copy-number gains and losses, the Genomic Identification of Significant Targets in Cancer (GISTIC, Broad Institute, Boston, USA) algorithm was utilized. Thresholds of LRR were set at 0.1 and −0.1 to allow GISTIC to identify amplifications and deletions, respectively. *Q*-values of minimal common regions <0.01 were defined as significant, and 0.99 was used as the confidence level to determine regions that contained potential driver genes. For genes within candidate CNV markers, the differential gene expression analysis was performed using NCBI GEO2R for the dataset GSE74385 in Gene Expression Omnibus (GEO) (29); the survival analysis for diverse tumors using the gene expression data and clinical information in The Cancer Genome Atlas (TCGA) project via the portal UALCAN (30).

### Statistics

Chi-squared tests and Wilcoxon rank-sum tests were performed using *R* scripts. The two-sided significance level was set at *p* ≤ 0.01, two-sided. Gene set enrichment analysis (GSEA) was performed using online tools (31), and the two-sided significance level was set at *q* ≤ 0.05. Prognostic analyses were conducted using Kaplan–Meier analysis and a Cox proportional regression model. Before conducting the prognostic analysis, patients with history of surgery, or with STR, or with postoperative radiotherapy were excluded. The two-sided significance level was set at *p* ≤ 0.05.

## Results

### Patient Characteristics

A total of 383 meningioma patients were enrolled in this study, with the female-to-male ratio at 2.24 (265:118), consistent with previous observation. Their average age was 49 years old (18 to 81 years old). According to the 2016 WHO meningioma grading classification, meningiomas of WHO grade I accounted for 86% (331/383) of tumors, among which 89 were from male patients. Meningiomas of WHO grade II constituted 12% (19 in females compared with 27 in males) of tumors, while meningiomas of grade III were only 1.6%, with 4 from females and 2 from males. The median of tumor diameter was 4.30 cm, and 239 meningiomas located in the skull base. In all, 338 patients had primary meningiomas and 45 patients suffered from tumor recurrence. GTR was achieved in 302 patients and STR in 81 patients; meanwhile, 118 patients had peritumoral brain edema, and 57 had bone invasion. For the pathological subtypes of these meningiomas, there were 140 transitional, 117 meningothelial, 66 fibrous, 18 atypical, 14 angiomatous, 6 anaplastic, 5 chondroid, 5 clear cell, and 12 other types. The summarized clinicopathological features are shown in Table 1.

### Landscape of CNVs in Meningiomas

**Figure 1:**
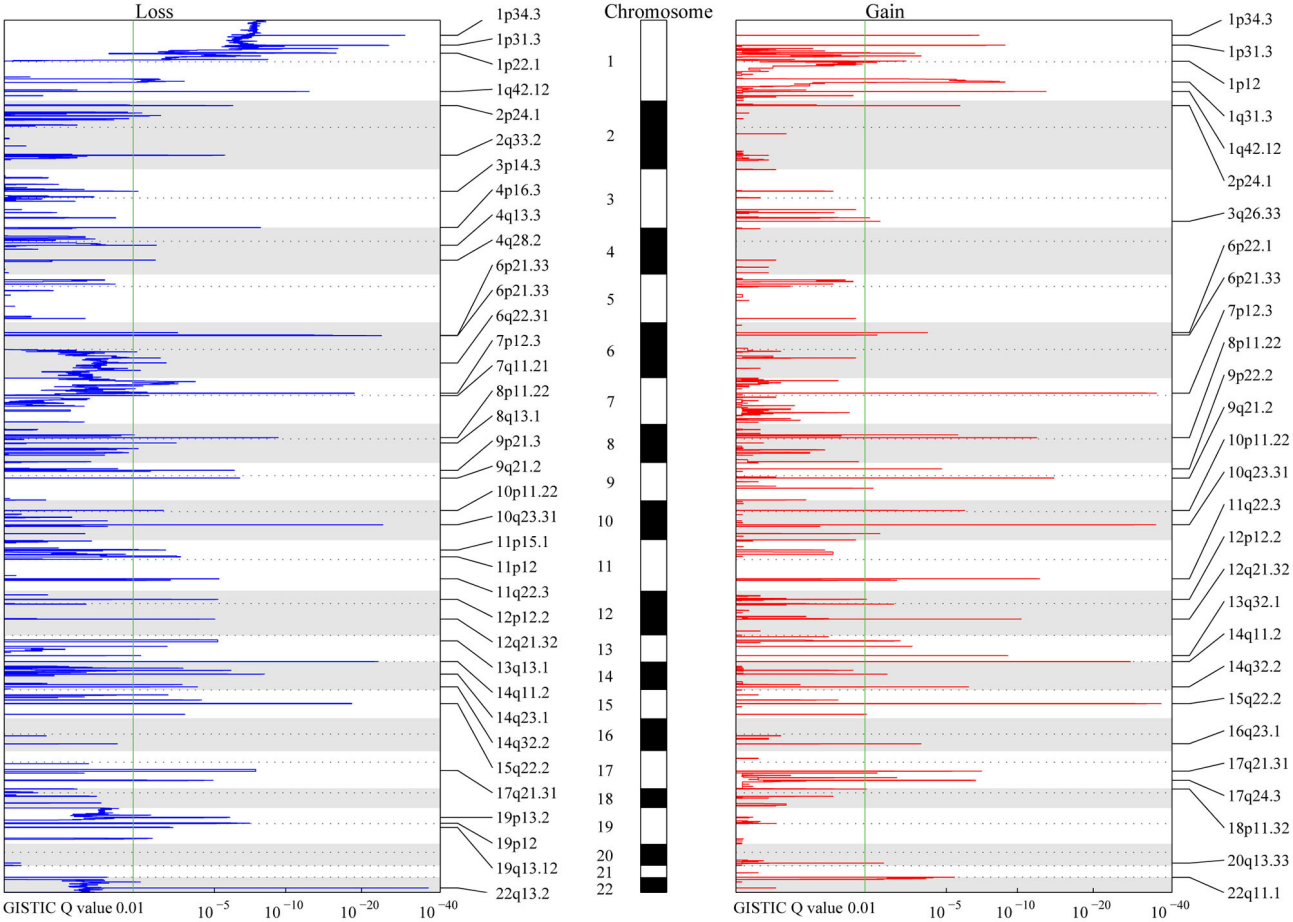
MCR Profiling of CNVs in meningiomas. The peaks in red and blue represent consistent regions of gains and losses in meningiomas based on Genomic Identification of Significant Targets in Cancer (GISTIC), respectively. The GISTIC *Q*-value is shown at the bottom. The green lines indicate the *Q*-value (0.01) considered significant in the analysis. Chromosomes are shown in the middle with odd-numbered chromosomes as white and even-numbered chromosomes as gray.

A total of 9,821 high-confidence CNVs were identified, and each sample had 26 CNVs on average, including 6,416 gains and 3,405 losses (detailed information for each CNV, Table S1). Their sizes ranged from 298 bp to 198 Mb, with 6,722 over 500 kb and 2,869 over 5 Mb. According to MCRs covered by diverse CNVs (Materials and Methods), we identified 36 common losses (39 kb to 79 Mb) in 27 chromosomes and 28 common gains (2 kb to 20 Mb) in 23 chromosomes in these 383 meningiomas (Figure 1 and Table 2). Copy-number losses (CNLs) were most likely to occur in 22q13.2 (31%), followed by 14q11.2 (29%). Moreover, chromosome 1 was also likely to lose fragments of its short arm, with three common losses of 1p34.3 (21%), 1p31.3 (19%), and 1p22.1 (16%). In comparison, copy-number gains (CNGs) were frequently detected at 22q11.1 (35%), 15q22.2 (16%), 14q11.2 (14%), 10q23.31 (14%), 8p11.22 (14%), and 7p12.3 (14%).

**Table 2:** List of MCRs of CNVs in meningiomas.

**Cytoband**	**CNV**	**Boundaries**	**Size (kb)**	**Number of genes**	**Frequency (%)**
1p12	Gain	chr1:117631472–121239762	3,608	29	2.87
1p31.3	Gain	chr1:62921155–63282308	361	3	10.44
1p34.3	Gain	chr1:35453772–35870648	417	4	10.44
1q31.3	Gain	chr1:196525315–199864159	3,339	22	11.75
1q42.12	Gain	chr1:225137135–225458504	321	1	12.01
2p24.1	Gain	chr2:20078572–20173435	95	3	6.01
3q26.33	Gain	chr3:180322742–180397085	74	2	4.96
6p21.33	Gain	chr6:31237665–31322196	85	2	8.09
6p22.1	Gain	chr6:29910981–29913077	2	1	6.79
7p12.3	Gain	chr7:48313001–48318810	6	1	13.58
8p11.22	Gain	chr8:38971607–39678651	707	6	13.58
9p22.2	Gain	chr9:17269438–17484335	215	1	6.27
9q21.2	Gain	chr9:79827895–80010024	182	1	9.66
10p11.22	Gain	chr10:32740815–33165314	424	1	5.22
10q23.31	Gain	chr10:91469746–91505720	36	1	13.58
11q22.3	Gain	chr11:103004336–103153757	149	1	10.70
12p12.2	Gain	chr12:21011481–21417898	406	4	11.23
12q21.32	Gain	chr12:88465703–88566416	101	3	12.01
13q32.1	Gain	chr13:96506648–96705463	199	1	10.70
14q11.2	Gain	chr14:1–20425050	20,425	16	13.58
14q32.2	Gain	chr14:96756059–96813532	57	1	7.05
15q22.2	Gain	chr15:62202415–62332979	131	1	16.45
16q23.1	Gain	chr16:75766089–76806431	1,040	1	6.27
17q21.31	Gain	chr17:41256213–41276031	20	1	11.75
17q24.3	Gain	chr17:66878095–67324998	447	7	10.97
18p11.32	Gain	chr18:2533131–2831495	298	4	7.83
20q13.33	Gain	chr20:58405221–58519202	114	4	8.09
22q11.1	Gain	chr22:1–18300886	18,301	27	35.25
1p22.1	Loss	chr1:93620394–94027864	407	4	15.67
1p31.3	Loss	chr1:62767954–63632517	865	4	19.06
1p34.3	Loss	chr1:35444038–35900519	456	5	20.63
1q42.12	Loss	chr1:224916594–225590674	674	1	11.75
2p24.1	Loss	chr2:20076455–20197016	121	2	6.27
2q33.2	Loss	chr2:203621938–204193687	572	5	10.18
3p14.3	Loss	chr3:57187079–57994564	807	8	5.22
4p16.3	Loss	chr4:1–493146	493	8	9.66
4q13.3	Loss	chr4:58064465–77139509	19,075	104	7.83
4q28.2	Loss	chr4:128748468–129193525	445	4	8.88
6p21.33	Loss	chr6:31324926–31467364	142	3	11.75
6p21.33	Loss	chr6:31239830–31368124	128	1	10.18
6q22.31	Loss	chr6:73934060–152442820	78,509	397	7.05
7p12.3	Loss	chr7:48147076–48981328	834	2	7.05
7q11.21	Loss	chr7:57531190–64451645	6,920	19	7.57
8p11.22	Loss	chr8:39142265–39771450	629	5	15.14
8q13.1	Loss	chr8:67577141–68113721	537	11	6.79
9p21.3	Loss	chr9:21865843–22447070	581	4	4.44
9q21.2	Loss	chr9:79783752–80039005	255	2	9.66
10p11.22	Loss	chr10:32634973–33190566	556	1	4.96
10q23.31	Loss	chr10:91405045–91592197	187	2	15.14
11p12	Loss	chr11:36680720–43605303	6,925	7	5.22
11p15.1	Loss	chr11:17062455–17408024	346	5	6.79
11q22.3	Loss	chr11:102935067–103734644	800	2	9.66
12p12.2	Loss	chr12:21069809–21417617	348	2	12.27
12q21.32	Loss	chr12:88439501–88890670	451	2	11.23
13q13.1	Loss	chr13:32886039–32977098	91	1	17.23
14q11.2	Loss	chr14:1–20443750	20,444	16	28.98
14q23.1	Loss	chr14:58734239–59101447	367	4	14.10
14q32.2	Loss	chr14:96731074–96846091	115	1	12.01
15q22.2	Loss	chr15:61509172–62359861	851	1	15.14
17q21.31	Loss	chr17:41180695–41278115	97	1	4.96
19p12	Loss	chr19:19906363–23958291	4,052	42	12.53
19p13.2	Loss	chr19:11842324–12757476	915	25	10.44
19q13.12	Loss	chr19:36950172–38319896	1,370	34	8.36
22q13.2	Loss	chr22:42518427–42557362	39	2	30.55

*MCRs, minimal common regions*.

**Figure 2:**
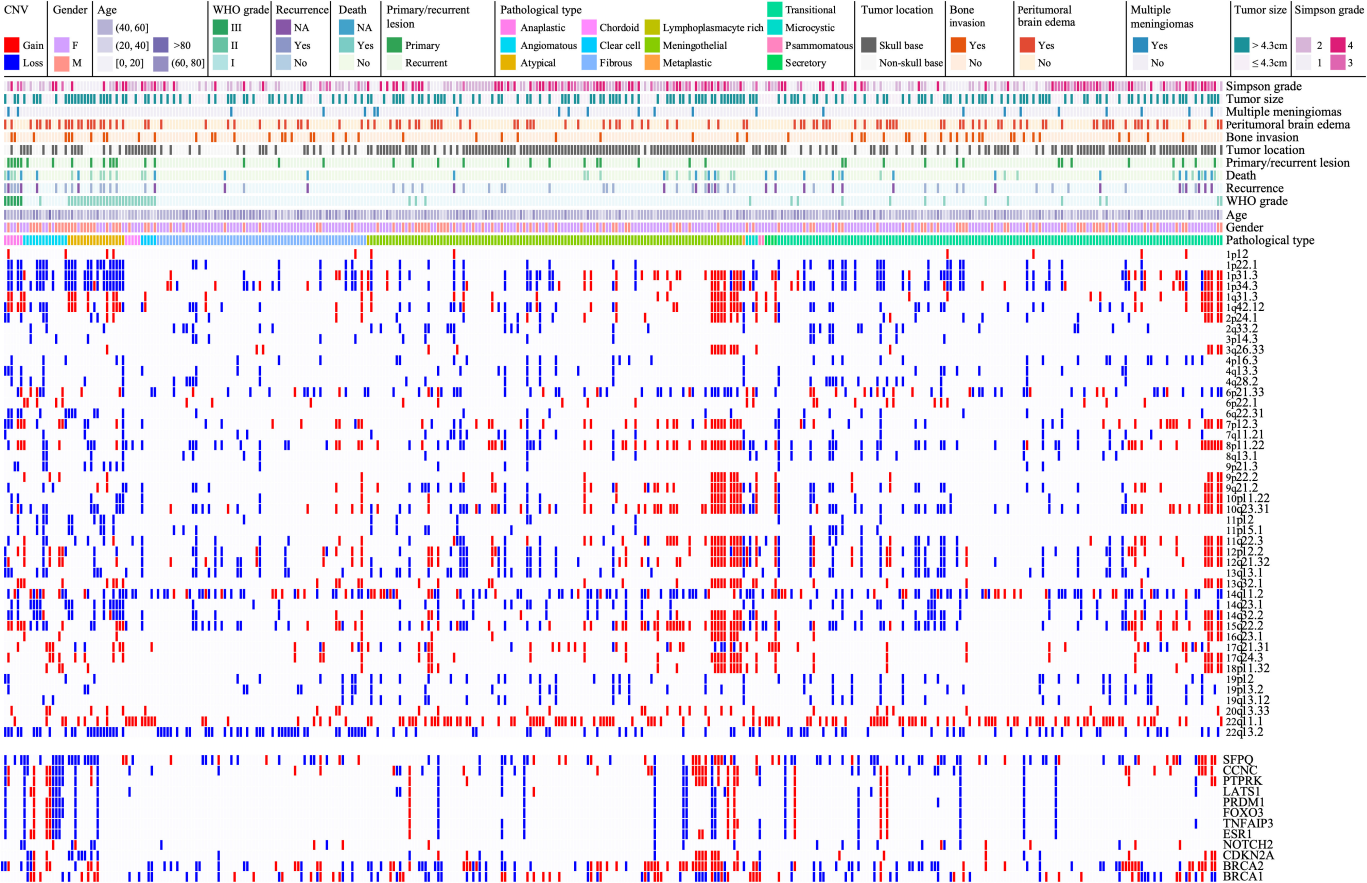
Landscape of CNVs in meningiomas. The solid bar in the same column represents a single MCR of a CNV with colors of red for gain, blue for loss, and white for normal. Different clinical information is represented at the top, which includes gender, age, WHO grade, death state, recurrent state, primary/recurrent lesions, pathology subtype, tumor location (skull base and non-skull base), bone invasion, peritumoral brain edema, multiple lesions, tumor size (>4.3 cm), and Simpson grade with colors as shown in the figure legends.

Particularly, four CNLs at 1p22.1, 8p11.22, 14q32.2, and 22q13.2 were more common in non-skull-base meningiomas, and a CNG at 22q11.1 more frequently occurred in skull-base meningiomas (Figure 2). These CNVs commonly led to the deletion of 737 genes and the amplification of 146 genes. Pathways over-represented by deleted genes were G2M checkpoint, IL6 JAK STAT3 signaling, TNFA signaling via NFKB, epithelial–mesenchymal transition, inflammatory response, and KRAS signaling (GSEA, Materials and Methods, Table S1).

### High CNV Burdens in Either WHO Grade II and III Meningiomas or Recurrent Lesions Featured by Large CNVs Over 500 kb

**Figure 3:**
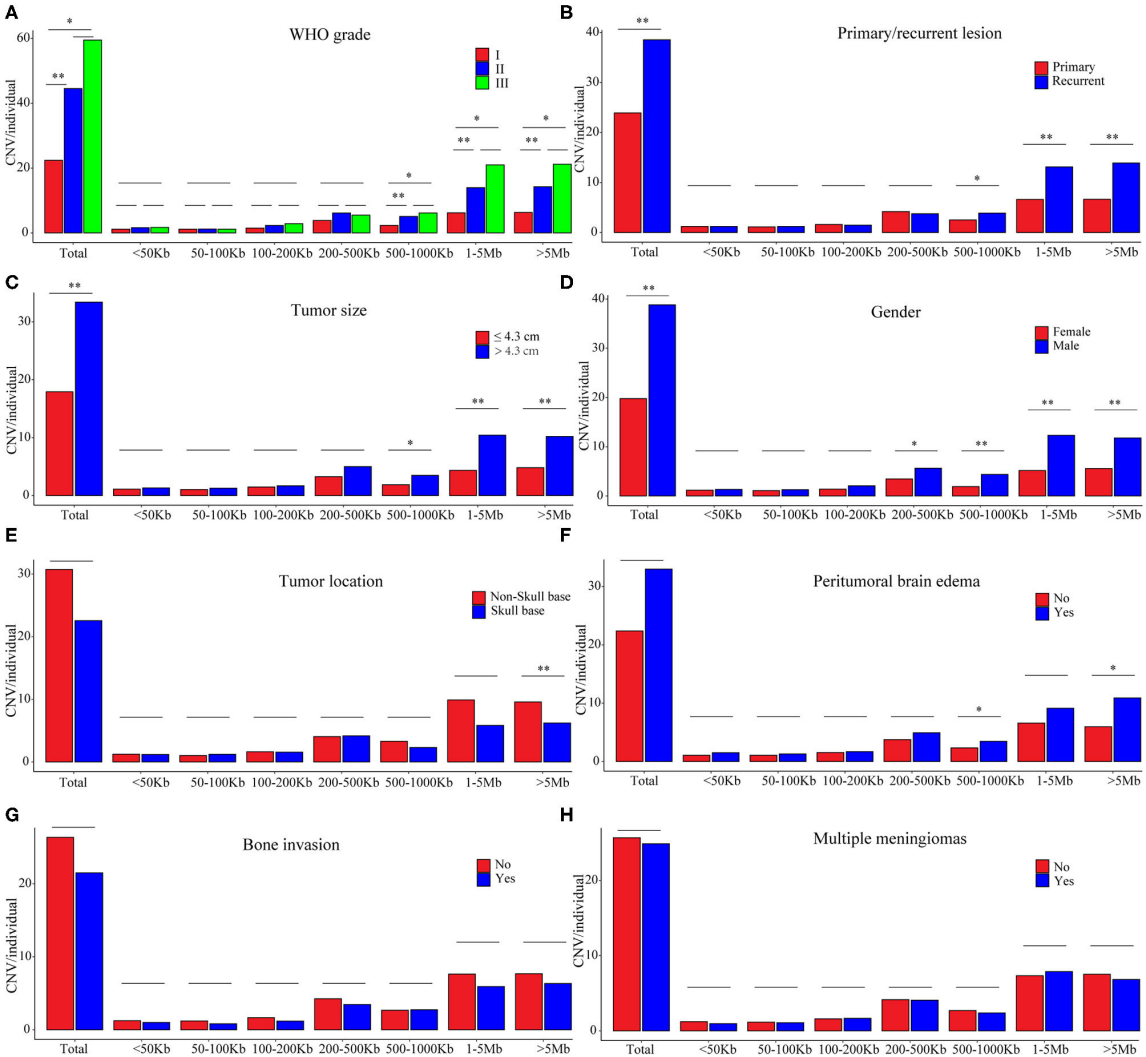
CNV burdens in meningiomas with different clinical features. *P*-values were calculated with Wilcoxon rank-sum test at each size range. *P < 0.01; **P < 0.001. **(A)** Average number of CNVs in meningiomas of different WHO grades. **(B)** Average number of CNVs in meningiomas of primary and recurrent lesions. **(C)** Average number of CNVs in meningiomas of different tumor sizes. **(D)** Average number of CNVs in meningiomas of different gender. **(E)** Average number of CNVs in meningiomas of different positions. **(F)** Average number of CNVs in meningiomas with or without peritumoral brain edema. **(G)** Average number of CNVs in meningiomas with or without brain edema. **(H)** Average number of CNVs in single or multiple lesions.

In the grade I meningiomas, 331 samples had 7,416 CNVs (22 per sample); in the 46 grade II meningiomas, there were 2,048 CNVs (45 per sample); and 6 grade III meningiomas had 357 CNVs (60 per sample). Therefore, the number of CNVs was similar in grade II and grade III meningiomas (II and III together defined as high grade, *p* = 0.374, Wilcoxon rank-sum test), which was significantly higher than that in grade I (*p* = 8.61 × 10^−4^ and 6.74 × 10^−3^, respectively, Wilcoxon rank-sum tests, Figure 3A). Moreover, the size of CNVs in high-grade meningioma was larger. The CNVs at 500–1,000 kb (5 vs. 6 vs. 2), 1–5 Mb (14 vs. 21 vs. 6), and >5 Mb (14 vs. 21 vs. 6) were more common in high-grade meningiomas than in grade I (*P* < 0.01, Wilcoxon rank-sum tests). Meanwhile, meningiomas at three grades had similar number of small CNVs (<500 kb) (grade I, 8; grade II, 11; and grade III, 11). Overall, large CNVs contributed to the higher CNV burden in WHO grade II and III meningiomas. In all, we found 15 common CNVs with differential incidences among different grades of meningiomas (Table 3). Interestingly, two CNLs at 4q13.3 and 6q22.31, both larger than 19 Mb, were most commonly observed in high-grade samples (*P* = 4.01 × 10^−7^, 4.05 × 10^−10^, respectively, Chi-square tests). A CNL at 4q13.3 covered 104 genes, which were over-represented in pathways including inflammatory response, IL6 JAK STAT3 signaling, TNFA signaling via NFKB, epithelial–mesenchymal transition, KRAS signaling up, and angiogenesis (Table S1). For 397 genes affected by the CNL at 6q22.31, the enriched pathways were Hypoxia, IL2 STAT5 signaling, and androgen response (Table S1).

**Table 3:** MCRs of CNVs differently distributed in meningiomas of different WHO grade, history of surgery, and tumor size.

**Cytoband**	**CNV**	**WHO grade**			***P***	**History of surgery**		***P***	**Tumor size**		***P***
		**I**	**II**	**III**		**Primary**	**Recurrent**		**Small**	**Large**	
1p22.1					9.42 × 10^−7**^			1.75 × 10^−6**^			
	Loss	40	16	4		42	18				
	No change	291	30	2		296	27				
1p31.3					1.90 × 10^−5**^			3.38 × 10^−6**^			2.10 × 10^−3*^
	Loss	50	19	4		52	21		26	47	
	No change	244	24	2		249	21		151	119	
	Gain	37	3	0		37	3		15	25	
1p34.3					9.30 × 10^−5**^			3.50 × 10^−7**^			9.65 × 10^−6**^
	Loss	56	18	4		55	23		25	53	
	No change	243	21	2		247	19		155	111	
	Gain	32	7	0		36	3		12	27	
1q31.3					2.20 × 10^−4**^						
	No change	300	35	3							
	Gain	31	11	3							
1q42.12					7.64 × 10^−6**^						
	Loss	36	8	1							
	No change	264	27	1							
	Gain	31	11	4							
2p24.1					1.88 × 10^−4**^						
	Loss	19	2	3							
	No change	294	39	3							
	Gain	18	5	0							
4q13.3					4.01 × 10^−7**^						
	Loss	22	4	4							
	No change	309	42	2							
6q22.31					4.05 × 10^−10**^			2.31 × 10^−5**^			
	Loss	15	8	4		17	10				
	No change	316	38	2		321	35				
7p12.3					3.49 × 10^−5**^						
	Loss	18	6	3							
	No change	266	37	1							
	Gain	47	3	2							
9p21.3					1.55 × 10^−5**^			1.16 × 10^−4**^			
	Loss	9	6	2		10	7				
	No change	322	40	4		328	38				
10p11.22					4.48 × 10^−3*^						
	Loss	12	5	2							
	No change	301	39	4							
	Gain	18	2	0							
11p12					1.86 × 10^−3*^			9.10 × 10^−4**^			
	Loss	12	7	1		13	7				
	No change	319	39	5		325	38				
14q23.1					4.00 × 10^−3*^			2.00 × 10^−3*^			
	Loss	39	13	2		41	13				
	No change	292	33	4		297	32				
14q32.2					2.44 × 10^−5**^						
	Loss	29	14	3							
	No change	277	30	3							
	Gain	25	2	0							
22q13.2					7.92 × 10^−4**^						
	Loss	91	21	5							
	No change	240	25	1							

MCRs, minimal common regions. P-values were calculated with chi-square tests. *P < 0.01; **P < 0.001. WHO grade was in accordance with 2016 WHO classification of meningiomas. Tumor size was dichotomized at median value of 4.30 cm. CNV, copy-number variation.

Recurrent meningiomas had significantly more CNVs (39 per sample) than primary ones (24 per sample, *p* = 2.08 × 10^−3^, Wilcoxon rank-sum test, Figure 3B). Meanwhile, recurrent lesions also had more CNVs over 500 kb (31 vs. 16, *p* = 3.03 × 10^−4^, Wilcoxon rank-sum test). Among seven common CNVs differentially distributed between recurrent and primary meningiomas (*P* < 0.01, chi-squared tests, Table 3), four CNVs of 1p31.3, 6q22.31, 9p21.3, and 11p12 were over 500 kb. Chromosome fragment losses at these sites were more common in recurrent meningiomas. For disrupted genes in each site (Sheet 3, Table S1), 6q22.31 covers nearly 400 genes. Other sites have much less genes affected, including four genes in 1p31.3 (*USP1, ANGPTL3, ATG4C*, and *DOCK7*), four in 9p21.3 (*CDKN2A, CDKN2B, C9orf53*, and *CDKN2B-AS1*), and seven in 11p12 (*API5, TTC17, LRRC4C, HNRNPKP3, MIR129-2, MIR670*, and *LOC100507205*).

### High CNV Burdens in Meningiomas of Large Diameter and Male Patients

According to the median of tumor diameter (4.30 cm), we grouped these tumors into large (>4.3 cm) and small groups (≤4.3 cm). The large group had 191 samples with 33 CNVs on average, significantly higher than the observation in the small group (192 samples with 18 CNVs on average, *P* = 7.51 × 10^−4^, Wilcoxon rank-sum test, Figure 3C). Besides, large meningiomas also had more CNVs over 500 kb (24 vs. 11, *p* = 4.84 × 10^−4^, Wilcoxon rank-sum test). Large lesions had more CNVs of 1p31.3 and 1p34.3, and only one CNV, either loss or gain, was over 500 kb locating at 1p31.3 (four genes affected, Table S1).

Meningiomas from male patients had significantly more CNVs (118 samples, 39 CNVs on average) than those in female patients (265 samples, 20 CNVs on average; *p* = 4.11 × 10^−6^, Wilcoxon rank-sum test, Figure 3D). Moreover, these CNVs in male samples were larger, more of which were over 200 kb (male: 34 vs. female: 16, *p* = 3 × 10^−6^, Wilcoxon rank-sum test). Seven common CNVs showed significant gender difference (*P* < 0.01, chi-squared tests, Table 4). Five out of them were over 200 kb including the CNVs of 1p22.1, 1p31.3, 1p34.3, 14q23.1, and 19p12. The CNLs at these sites were more common in meningiomas from male patients. For genes affected by these CNVs (Table S1), there were four located in 1p22.1 (*DR1, FNBP1L, CCDC18*, and *LOC100131564*), four in 1p31.3 (*USP1, ANGPTL3, ATG4C*, and *DOCK7*), four in 14q23.1 (*ARID4A, KIAA0586, TIMM9*, and *TOMM20L*), five in 1p34.3 (*SFPQ, ZMYM4, ZMYM6, ZMYM1*, and *ZMYM6NB*), and 42 in 19p12.

**Table 4:** MCRs of CNVs differently distributed in meningiomas of different gender, tumor location, and with or without peritumoral brain edema.

**Cytoband**	**CNV**	**Gender**		***P***	**Tumor location**		***P***	**PBE**		***P***
		**F**	**M**		**Non-skull base**	**Skull base**		**No**	**Yes**	
1p22.1				4.55 × 10^−4**^			2.00 × 10^−3*^			1.37 × 10^−3*^
	Loss	30	30		33	27		31	29	
	No change	235	88		111	212		234	89	
1p31.3				6.98 × 10^−4**^						
	Loss	37	36							
	No change	198	72							
	Gain	30	10							
1p34.3				1.38 × 10^−5**^						
	Loss	37	41							
	No change	201	65							
	Gain	27	12							
4p16.3										4.41 × 10^−3*^
	Loss							18	19	
	No change							247	99	
6p21.33				1.77 × 10^−3*^						
	Loss	50	20							
	No change	204	81							
	Gain	11	17							
8p11.22							2.20 × 10^−3*^			
	Loss				28	30				
	No change				107	166				
	Gain				9	43				
9p21.3										1.96 × 10^−3*^
	Loss							6	11	
	No change							259	107	
14q23.1				5.23 × 10^−9**^						7.83 × 10^−3*^
	Loss	19	35					29	25	
	No change	246	83					236	93	
14q32.2				1.77 × 10^−4**^			2.15 × 10^−4**^			
	Loss	20	26		27	19				
	No change	223	87		114	196				
	Gain	22	5		3	24				
19p12				6.05 × 10^−3*^						
	Loss	25	23							
	No change	240	95							
20q13.33										6.07 × 10^−4**^
	No change							252	100	
	Gain							13	18	
22q11.1							3.45 × 10^−5**^			
	No change				112	136				
	Gain				32	103				
22q13.2							4.63 × 10^−7**^			
	Loss				66	51				
	No change				78	188				

MCRs, minimal common regions. P-values were calculated with chi-square tests. ^*^P < 0.01; ^**^P < 0.001. CNV, copy-number variation; M, male; F, female; PBE, peritumoral brain edema.

### The Number of CNVs in Meningiomas Was Independent of Tumor Locations, Peritumoral Brain Edema, Bone Invasion, and Single or Multiple Lesions

Skull-base meningiomas (239 samples, 23 CNVs per sample) had a similar number of CNVs to with non-skull-base lesions (144 samples, 31 CNVs per sample; *p* = 0.013, Wilcoxon rank-sum test, Figure 3E). Overall, extremely large CNVs (>5 Mb) were more likely to present in non-skull-base meningiomas (*p* = 7.18 × 10^−5^, Wilcoxon rank-sum test). Nevertheless, a CNG at 22q11.1 of this type was preponderant in skull-base meningiomas (*p* = 3.45 × 10^−5^, chi-squared test, Table 4), affecting 27 genes in the region (Table S1).

Patients present with peritumoral brain edema (118 samples, 33 CNVs on average) showed no significantly difference in number of CNVs with those without (265 samples, 22 CNVs on average; *P* > 0.01, Wilcoxon rank-sum test, Figure 3F). However, more large CNVs (500 kb−1 Mb and >5 Mb) were observed in meningiomas with peritumoral brain edema (*P* = 3.48 × 10^−3^, 5.29 × 10^−3^, respectively, Wilcoxon rank-sum tests). The featured one with most significance located at 9p21.3, which was a CNL covering four genes (*CDKN2A, CDKN2B, C9orf53*, and *CDKN2B-AS1*) (*p* = 1.96 × 10^−3^, chi-square test; Table 4 and Table S1).

In tumors with or without bone invasions (*P* = 0.597) or single or multiple lesions (*P* = 0.869), the CNV burdens were similar (Wilcoxon rank-sum tests; Figures 3G,H). No CNVs were more prevalent in meningiomas with bone invasions. Nevertheless, a CNG at 10q23.31 had a higher incidence in multiple lesions (multiple, 33% vs. single, 12%; *p* = 3.49 × 10^−3^, chi-squared test, Table 5), which only covers one gene *KIF20B*.

**Table 5:** The MCRs of CNVs differently distributed in meningiomas of single or multiple lesions.

**Cytoband**	**CNV**	**Meningiomas**		***P***
		**Single**	**Multiple**	
10q23.31				3.49 × 10^−3^*
	Loss	58	0	
	No change	257	16	
	Gain	44	8	

MCRs, minimal common regions. P-values were calculated with chi-square tests. *P < 0.01. CNV, copy-number variation.

### Identification of Independently Significant Prognostic CNVs in Predicting Tumor Recurrence

**Figure 4:**
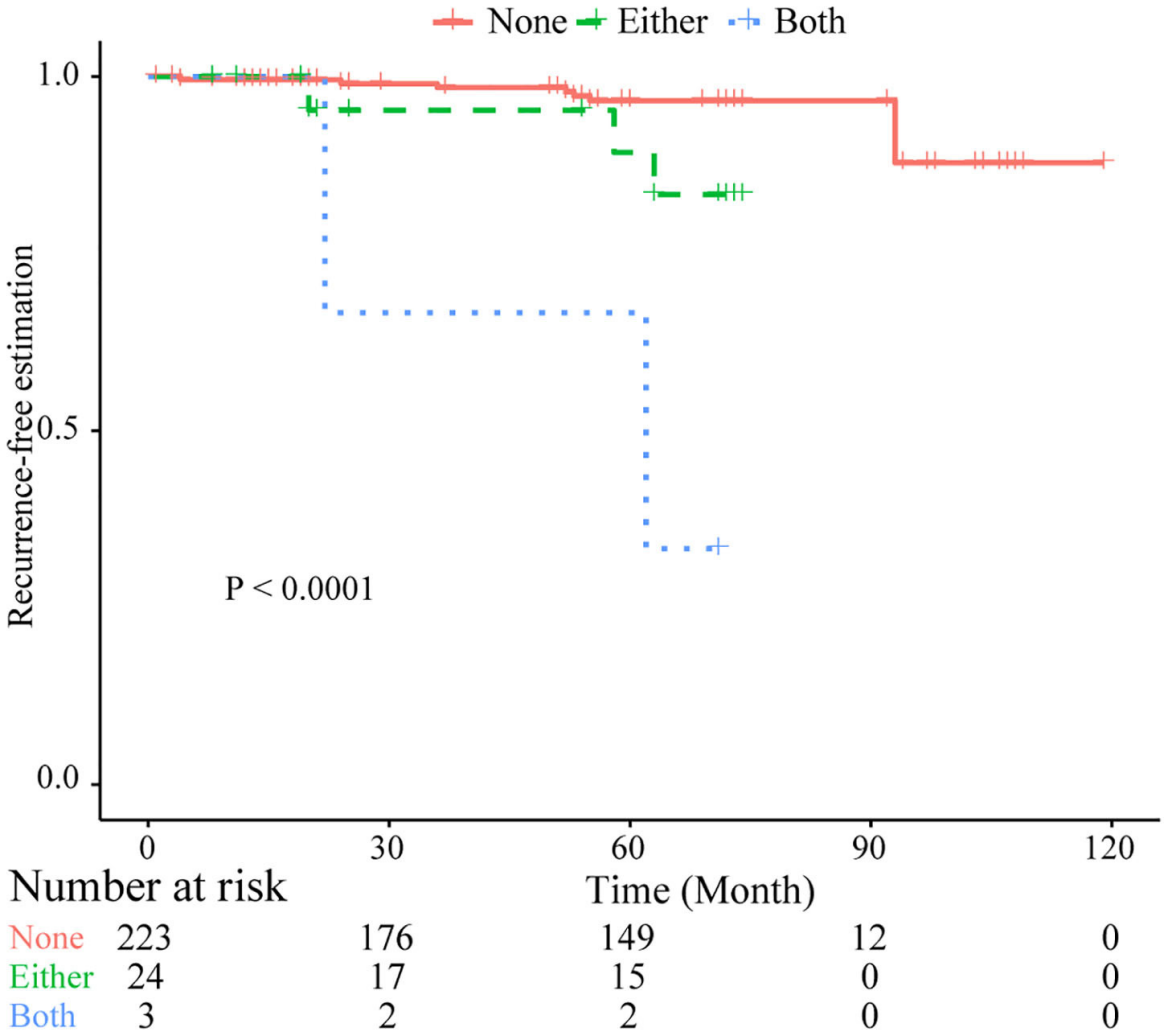
Kaplan–Meier plots of combined losses involving the CNLs at 4p16.3 and 10p11.22 as risk factor of tumor recurrence. Log-rank test was used. Both, with CNLs identified at both sites; Either, with CNLs identified at only one of two sites; None, no CNLs at both sites.

Based on common CNVs, we tried to predict the tumor recurrence. After excluding patients with recurrent lesions, with subtotal resection, or having postoperative radiotherapy, 267 patients were included for further prognostic analysis, and the detailed clinicopathological features of this subcohort are shown in Table 1. In the follow-up (mean period, 60 months), 12 patients suffered from tumor recurrence. All common CNV regions and clinical features were included in univariate Cox analysis of tumor recurrence. As shown in Table 6, skull-base lesions (*p* = 0.040), loss of 1p22.1 (*p* = 0.039), 1p34.3 (*p* = 0.024), 4q13.3 (*p* = 0.029), 4p16.3 (*p* = 0.001), 7q11.21 (*p* = 0.015), 10p11.22 (*p* = 0.003), 14q23.1 (*p* = 0.032), 19q13.12 (*p* = 0.013), and 19p12 (*p* = 0.01) were significant risk factors for tumor recurrence. In particular, most significant independent risk factors for recurrence were loss of 4p16.3 (*p* = 0.009, HR = 5.69, multivariate Cox analysis) and 10p11.22 (*p* = 0.037, HR = 4.53). As shown in Figure 4, patients with losses of both 4p16.3 and 10p11.22 were more likely to suffer from tumor recurrence than patients with loss of either one, or patients with neither of these CNV changes. Calculated by Cox analysis, the hazard ratio (HR) increased by 5.10 (95% CI: 2.35–11.08, *p* = 3.7 × 10^−5^) for each additional prognostic CNV.

**Table 6:** Significant factors for tumor recurrence of meningiomas in subcohort for prognostic analysis.

	**Risk factor**		**P**	**HR**	**95% CI**	
**Univariate cox analysis**						
Tumor location	skull base	Yes vs. no	0.040	4.99	1.08	23.11
1p22.1	Loss	Yes vs. no	0.039	3.65	1.07	12.47
1p34.3	Loss	Yes vs. no	0.024	3.92	1.20	12.85
4q13.3	Loss	Yes vs. no	0.029	4.38	1.16	16.51
4p16.3	Loss	Yes vs. no	0.001	7.96	2.33	27.22
7q11.21	Loss	Yes vs. no	0.015	5.18	1.37	19.52
10p11.22	Loss	Yes vs. no	0.003	7.48	1.98	28.20
14q23.1	Loss	Yes vs. no	0.032	3.83	1.12	13.09
19q13.12	Loss	Yes vs. no	0.013	5.39	1.43	20.33
19p12	Loss	Yes vs. no	0.010	5.05	1.48	17.26
**Multivariate cox analysis**						
4p16.3	Loss	Yes vs. no	0.009	5.69	1.53	21.13
10p11.22	Loss	Yes vs. no	0.037	4.53	1.10	18.67

*HR, hazard ratio; CI, confidence interval*.

Eight genes located within these two CNLs: *ZNF141, ABCA11P, ZNF595, ZNF721, ZNF718, ZNF876P, ZNF732* in 4p16.3, and *CCDC7* in 10p11.22 (Table S1). In differential gene expression analysis between non-recurrent (grade I, 13 and grade II, 6) and recurrent (grade I, 7 and grade II, 8) lesions in a public gene expression dataset of meningioma (Method), *ZNF141* and *ZNF595* showed the tendency to have lower expression levels in recurrent samples (unadjusted *p* < 0.05, Table S1). We further examined the effects of expression levels of these eight genes on survival time for 31 tumor types in TCGA (Method) and identified 28 associations with significance (*p* < 0.05, Figure S1). All of these genes had lower expression levels in certain tumor types from patients with shorter survival, which indicated their decreased functions related to malignant phenotypes. Particularly, most of these genes (five out of eight) had the same effects on patient survival in head and neck squamous cell carcinoma (HNSC), and the low expression of *ZNF718* (*p* = 0.0027), *CCDC7* (*p* = 0.01), *ZNF141* (*p* = 0.012), *ZNF721* (*p* = 0.029), and *ZNF732* (*p* = 0.045) all demonstrated significant associations with the shorter survival of patients.

## Discussion

In the present study, we clarified the CNV characteristics of cranial meningiomas in 383 Chinese patients. Particularly, we compared the CNV burdens of meningiomas in diverse phenotypes. We found more CNVs in the samples of high-grades, recurrent lesions, tumor diameter over 4.3 cm, and samples from male patients. Meanwhile, CNV burden may not relate to tumor locations, peritumoral brain edema, bone invasion, and multiple lesions. Moreover, we also identified featured CNVs in each clinical group. Besides, we found two candidates as independent prognostic CNVs in predicting tumor recurrence.

Based on a relatively large cohort of cranial meningiomas, we observed that CNVs of 22q (61%), 14q (54%), and 1p (38%) were the most prevalent, followed by 15q (32%), 6p (30%), 8p (29%), 10q (29%), and 1q (25%). In previous studies, CNVs of 22q, 14q, and 1p are always among the most frequent CNVs of meningiomas (7, 10, 12, 14, 15, 24, 32), which is in accordance with our observation. For instance, a recent study reported the top three CNVs in their samples located at 1p (71%), 22q (64%), and 14q (42%) (14). By far, no differences were noted among different ethnic groups.

The CNVs frequently identified in patients with distinct clinical features hold clues for further functional studies. For instance, two CNLs of 1p31.3 and 1p34.3, commonly seen in meningiomas of high-grade and recurrent or large lesions, contain a lot of genes with functional importance. At 1p34.3, the *SFPQ* gene participates in transcriptional regulation, DNA double-strand break repairs, and suppression of RNA:DNA-hybrid-related telomere instability (33, 34). At 1p31.3, the *USP1* gene, involved in multiple DNA repair pathways, can function as a key senescence regulator controlling genomic integrity (35); autophagy protein *ATG4C* participates in controlling the unregulated cell growth (36). Reduced levels of autophagy have been described as being linked to malignant tumors (37). Functional changes related to these genes may also contribute to the progression of meningiomas, which needs further studies for validation.

The CNG at 10q23.31 was the only CNV more commonly seen in multiple meningiomas rather than in single lesions. It covers only one gene, *KIF20B*, an oncogene involved in cytokinesis. A recent study suggested to target the *KIF20B* gene in the treatment for hepatocellular carcinoma (38). Inhibition of KIF20B can block mitosis at both metaphase and telophase, which enhance the cytotoxicity of two chemotherapeutic drugs, hydroxycamptothecin, and mitomycin C (39). The role of *KIF20B* in tumorigenesis of meningiomas, especially multiple lesions, suggests that its suppression might be a novel strategy in the treatment for multiple meningiomas in the future. Moreover, the CNG at 6p21.33, more frequently found in lesions from male patients, is where *HLA-B* and *HLA-C* are located, indicating the existence of immune factors underlying gender difference of meningioma occurrence; the CNG at 20q13.33, more frequently identified in patients with peritumoral brain edema, covers the *SYCP2* gene, which is related to the depth of cervical invasion in squamous cell carcinoma (40).

Tumor recurrence is an important issue for patients with meningiomas, and patients with meningiomas prone to recurrence need adjuvant radiation therapy or close follow-up. Meanwhile, patients with low risk of tumor recurrence could be spared from the toxicity of radiation therapy. Nevertheless, these patients are not accurately identified by WHO grading (41). Here, we demonstrated the potential of CNV profiling in recurrence prediction. Loss of 1, 4, 9, and 10p and gain of 1q or other chromosomal regions have been revealed to be risk factors for tumor recurrence in previous studies (7–11, 14, 21, 24). In our observation, the CNLs of 4p16.3 and 10p11.22 were independent risk factors for cranial meningioma recurrence. The CNL of 4p16.3 covers MiR-571, *ABCA11P, ZNF141, ZNF595, ZNF718, ZNF721, ZNF732*, and *ZNF876P*. A recent study identified miR-571 as the first miRNA that prevents aberrant DNA replication, and the *Cdk2-c-Myc*-miR-571 axis was identified as a new pathway for regulating DNA replication, cell cycle, and genomic stability in cancer cells (42). As a result, loss of miR-571 may lead to genomic instability. Although some studies have reported differential expression or mutation occurrence of *ZNF595* (43), *ZNF721* (44), *ZNF718* (45), and *ZNF141* (46), their functions remain unclear. Besides, potential roles of *ABCA11P, ZNF732*, and *ZNF876P* are novel in meningioma recurrence. In the 10p11.22, CCDC7, also known as Biot2, highly expressed in CD133-positive stem cells, functions as a risk factor for poor prognosis in colorectal cancer (47, 48). In our study, the CNL at 10p11.22 (*CCDC7*) was an independent risk factor of tumor recurrence, and the underlying mechanisms need further investigation.

The cross-sectional analysis in the entire cohort compared primary and recurrent lesions from different groups of patients, and some primary tumors may also harbor CNVs contributing to tumor recurrence. It may undermine the ability to identify CNVs related to recurrence, which may explain the missing of the CNLs of 4p16.3 and 10p11.22 in the comparison. Meanwhile, the comparison results may be cofounded by differential CNVs present in the early stage of tumor development between two groups of patients. Therefore, the follow-up study provides us an opportunity to identify those CNVs related to recurrence. The recurrence rates of patients with these two CNVs were over 20% (loss of 4p16.3, 21%, 4/19; loss of 10p11.22, 27%, 3/11), significantly higher than the recurrent rate (about 4%) in patients without them. Nevertheless, only 12 patients (4.5%, 12/267) had tumor recurrence during a mean follow-up period of 5 years in our subcohort for prognostic analysis. Although it is similar to previous observation, which is 3% for WHO grade I meningiomas and 30% for WHO grade II meningiomas in GTR patients (28), the prediction power of these two candidate markers requires further evaluation in a larger group of patients with tumor recurrence in the future follow-up. Besides, the recurrence factors may have heterogeneity, and 4p16.3 and 10p11.22 together accounted for the 13% (loss of 4p16.3, 4/45; loss of 10p11.22, 4/45, losses of both, 2/45) recurrent lesions in the cross-sectional analysis. It needs further efforts to dissect other CNVs related to tumor recurrence.

## Conclusions

Based on a large number of patients with cranial meningiomas, we identified that the CNVs of 22q, 1p, and 14q were the most prevalent. Meningiomas of high WHO grades, recurrent tumors, large size, and male gender were likely to have more CNVs, especially of large size (>500 kB). Additionally, the CNLs at 4p16.3 and 10p11.22 were promising candidates as independent risk factors for tumor recurrence prediction.

## Data Availability Statement

The datasets in this study have been publicly deposited. They can be accessed at: https://www.ncbi.nlm.nih.gov/geo/query/acc.cgi?acc=GSE147673.

## Ethics Statement

The studies involving human participants were reviewed and approved by Institutional Review Board of Beijing Tiantan Hospital Affiliated with Capital Medical University. Signed informed consent forms were acquired from all patients or their guardians.

## Author Contributions

LW, DZ, JM, YH, ZW, JZ, LZ, and WC designed this study. JM, YH, KT, DL, KW, YY, and JD conducted the experiments and acquired data. JM, YZ, YC, LS, and LC conducted the follow-up and acquired data. JM and YH analyzed and interpreted data. JM, YH, KT, DL, KW, YY, JD, YZ, YC, LS, and LC drafted the manuscript. LW, DZ, ZW, JZ, WC, and LZ revised the manuscript. All authors have seen and approved the manuscript.

## Conflict of Interest

The authors declare that the research was conducted in the absence of any commercial or financial relationships that could be construed as a potential conflict of interest.
