# Supplementary material for: High Copy-Number Variation Burdens in Cranial Meningiomas From Patients With Diverse Clinical Phenotypes Characterized by Hot Genomic Structure Changes
Source: Front Oncol. 2020 Aug 14;10:1382. doi: 10.3389/fonc.2020.01382 (PMC7457130; doi:10.3389/fonc.2020.01382)

Supplementary Material

# Supplementary Table

Table S1.xlsx

Sheet 1. Number of CNV segments for each individual in different size ranges.

Sheet 2. Consistent genomic regions of amplification and related genes in meningiomas.

Sheet 3. Consistent genomic regions of deletion and related genes in meningiomas.

Sheet 4. Enriched gene sets revealed by Gene set enrichment analysis performed on affected genes.

Sheet 5. Differential gene expression analysis between non-recurrent and recurrent lesions in GSE74385 dataset in NCBI GEO database.

# Supplementary Figures

Figure S1 Survival analysis of TCGA datasets for genes at CNLs 4p16.3 and 10p11.22

1. Head and Neck squamous cell carcinoma (HNSC)


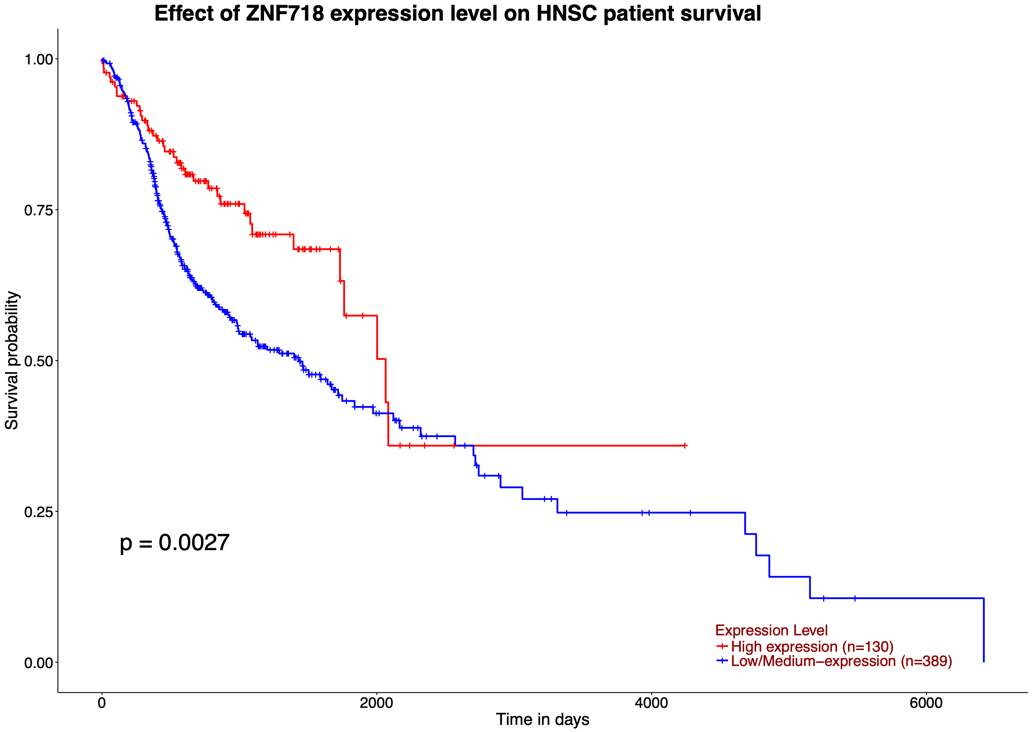

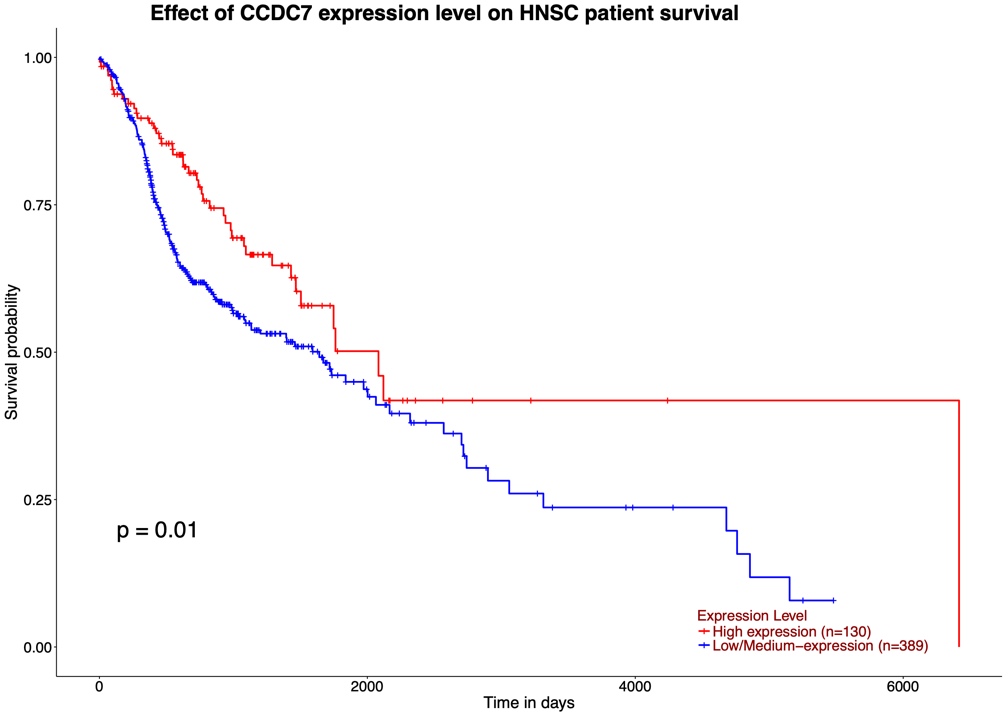


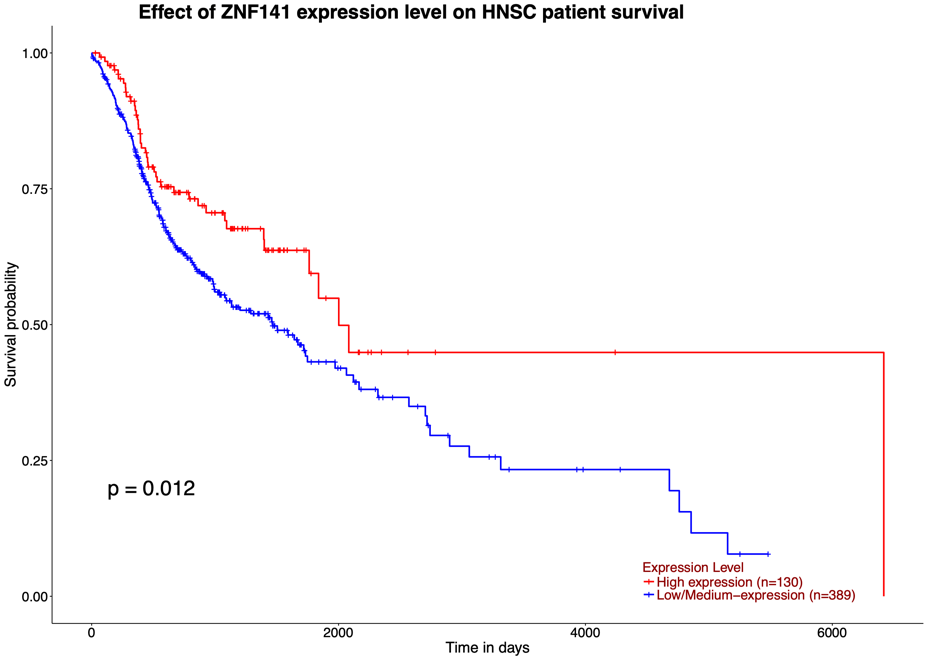


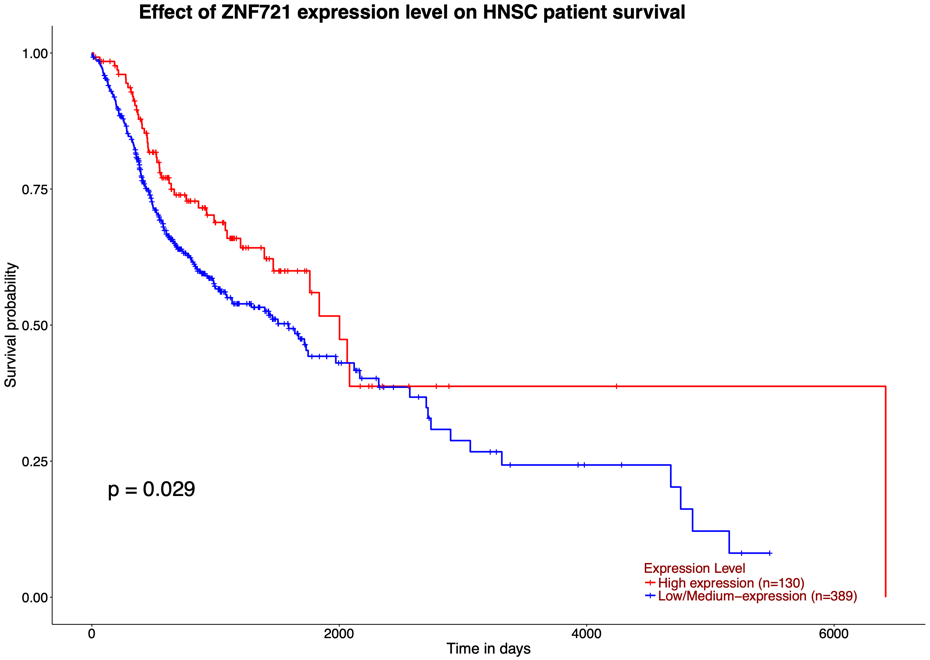

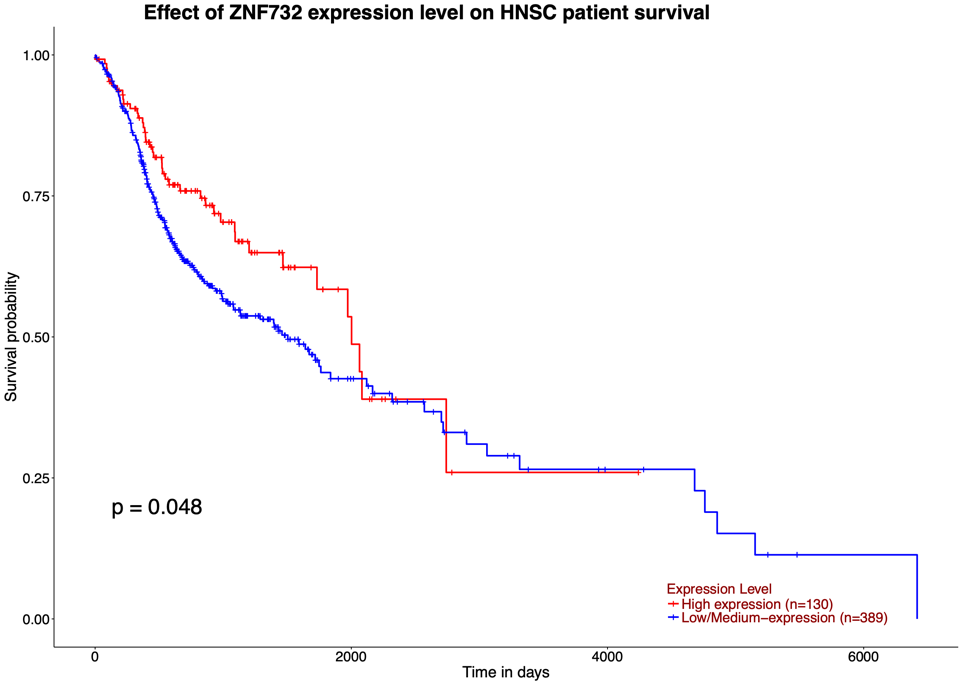


1. Kidney renal clear cell carcinoma (KIRC)


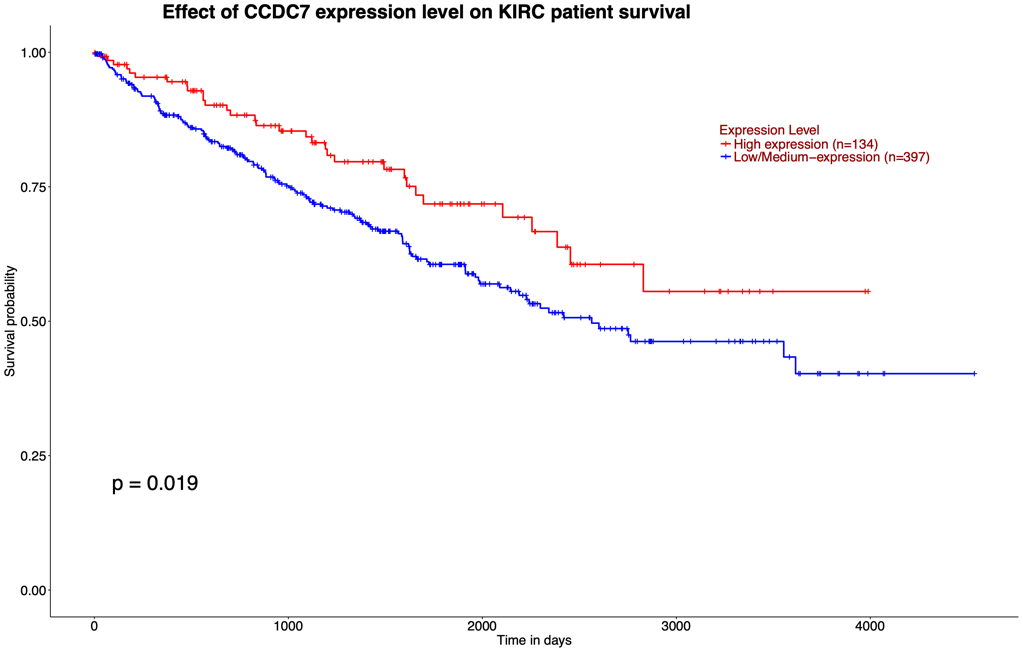


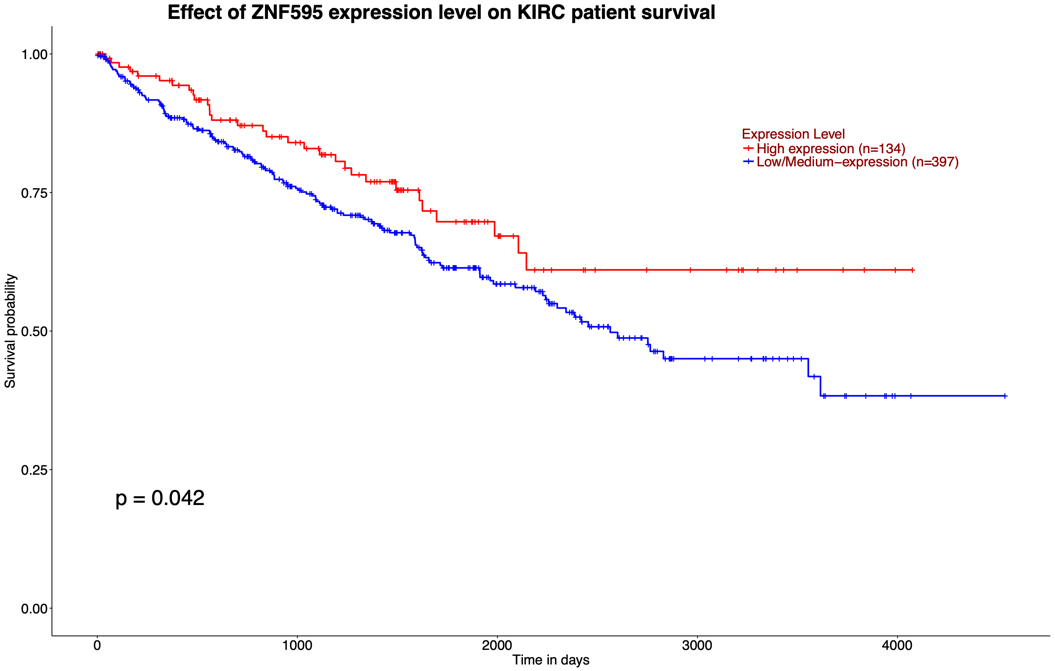


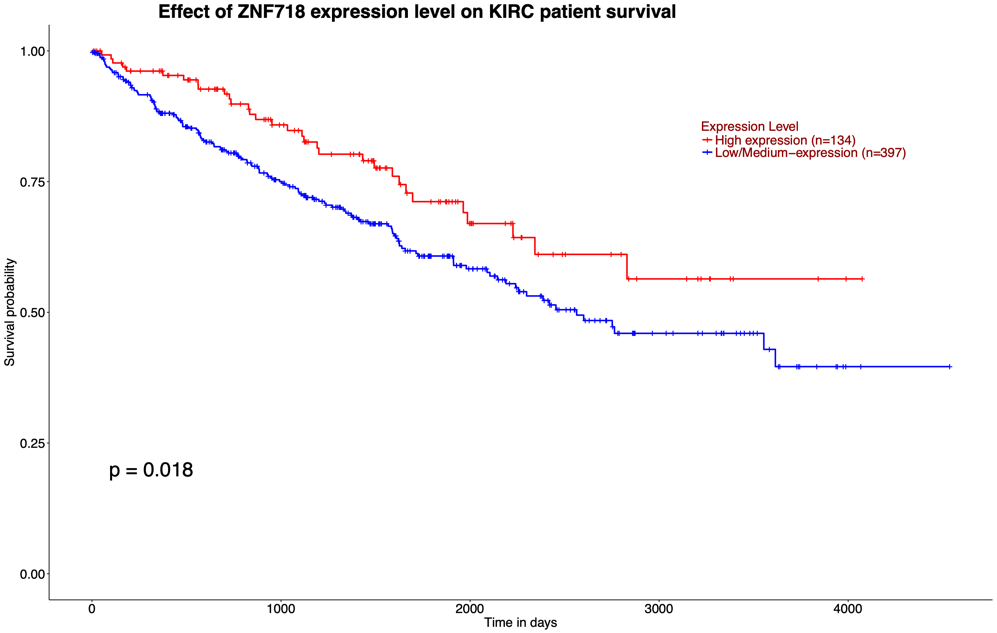


1. Skin Cutaneous Melanoma (SKCM)


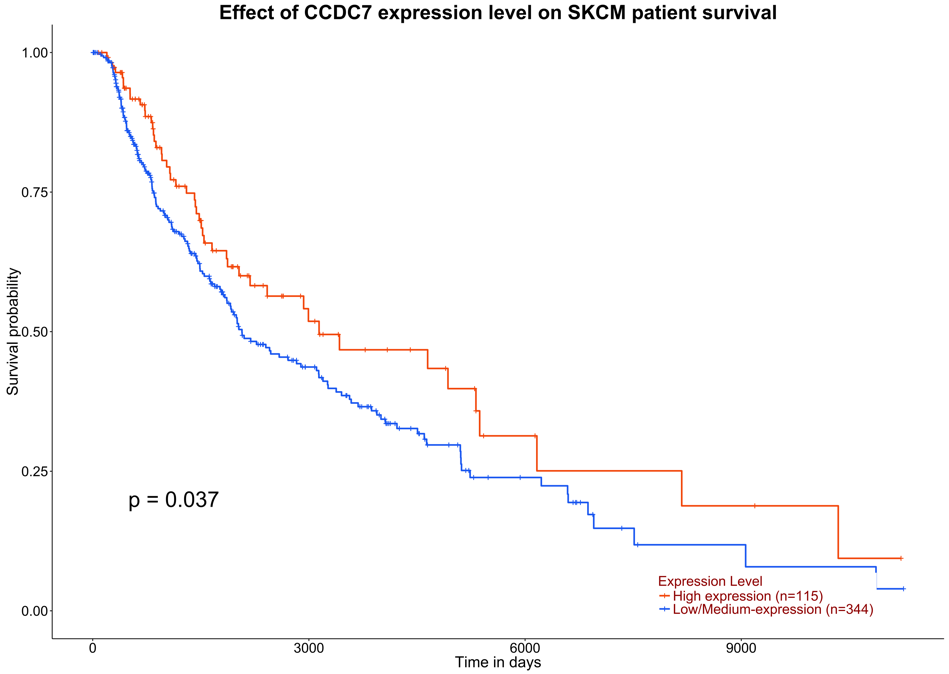


1. Glioblastoma multiforme (GBM)


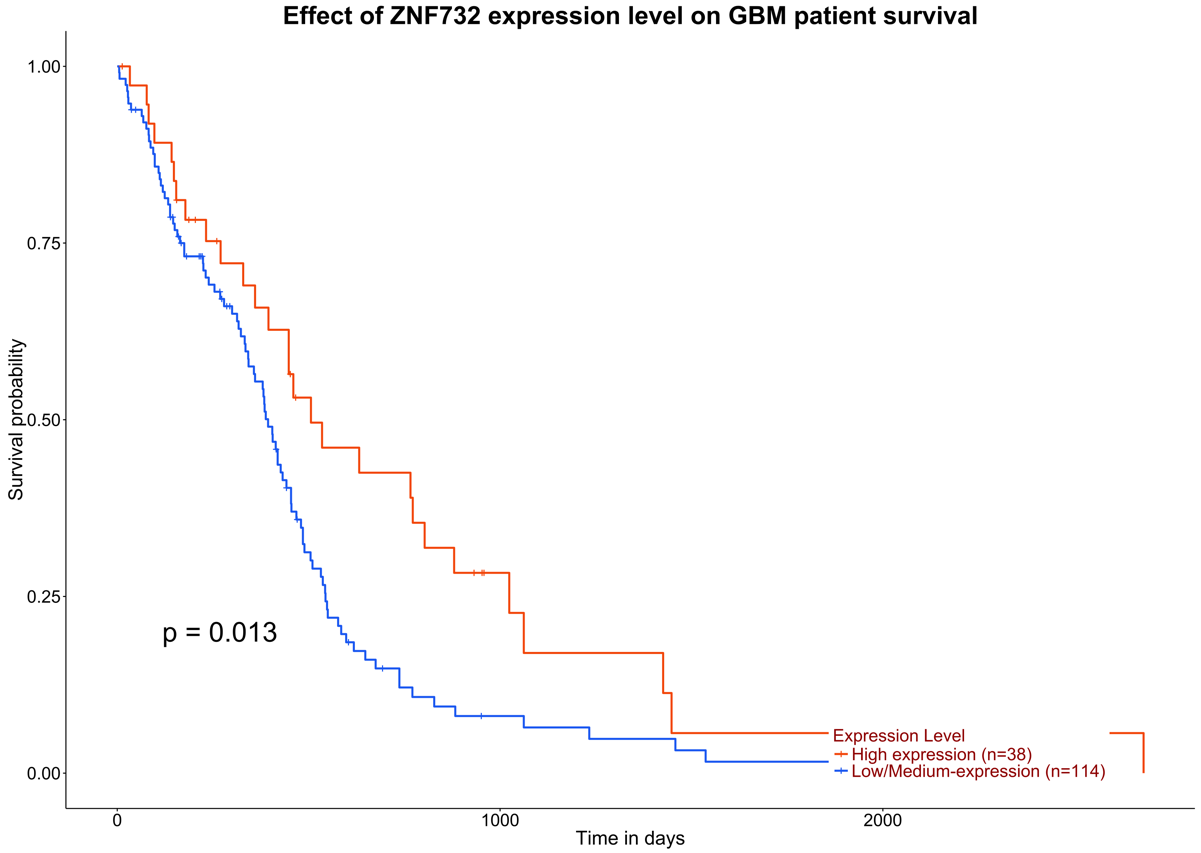


1. Uveal Melanoma (UVM)


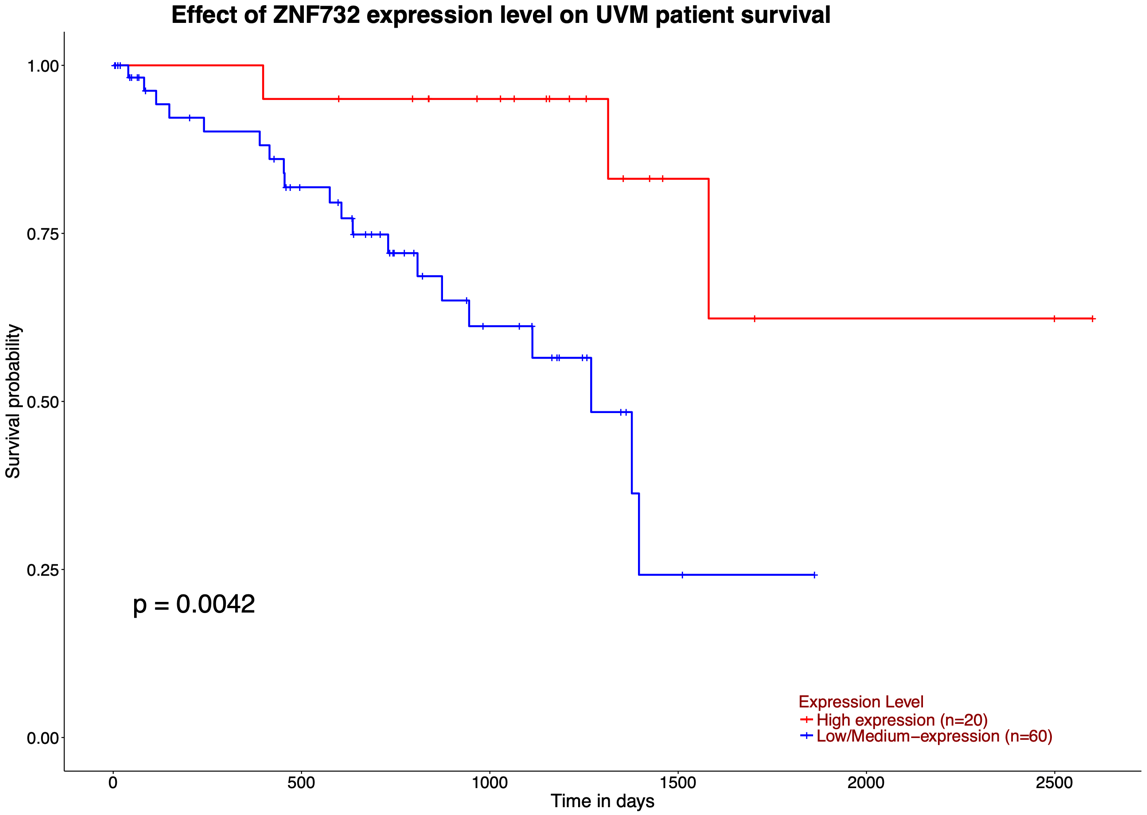


1. Ovarian serous cystadenocarcinoma (OV)


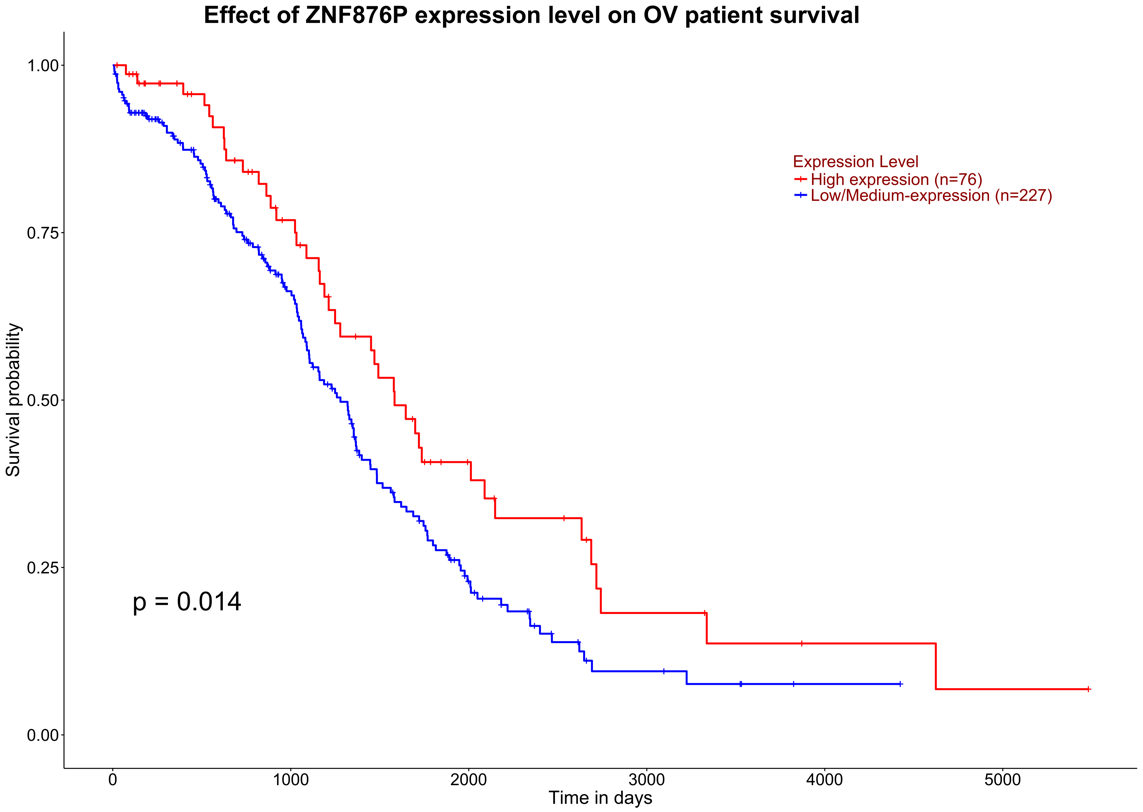


1. Pancreatic adenocarcinoma (PAAD)


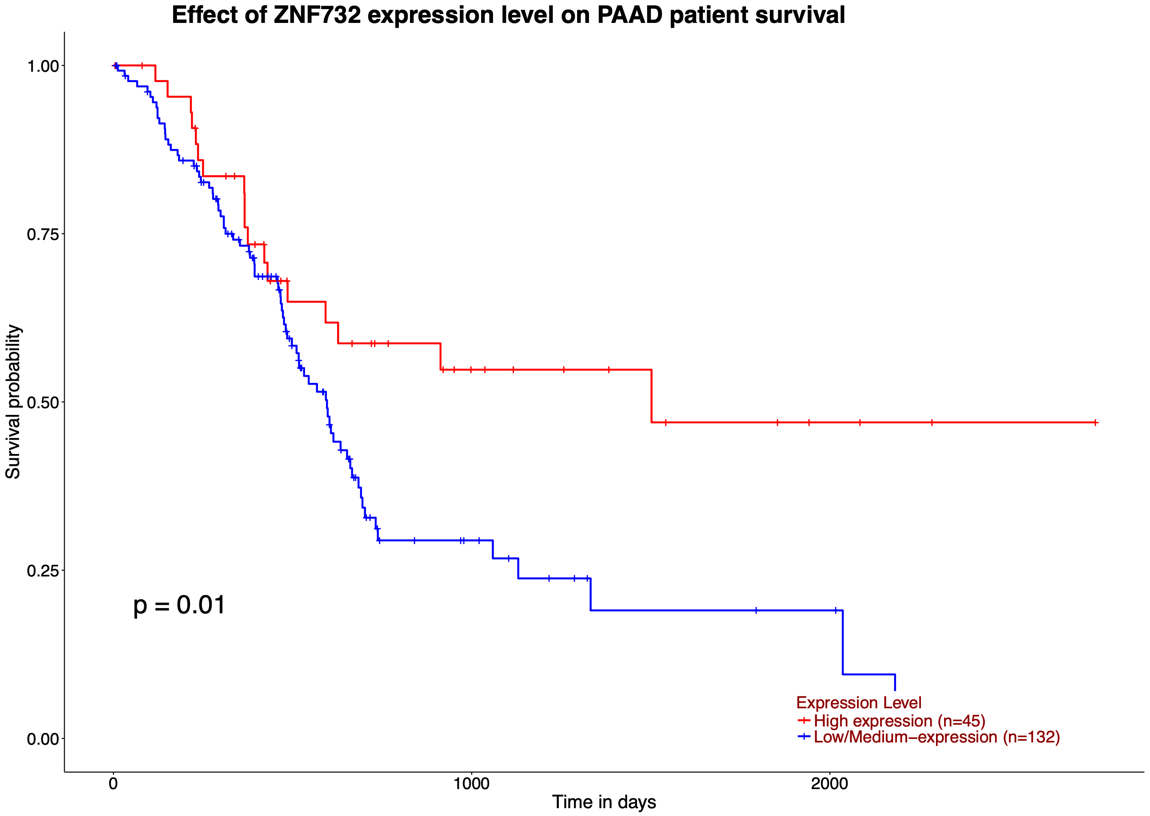


1. Bladder urothelial carcinoma (BLCA)


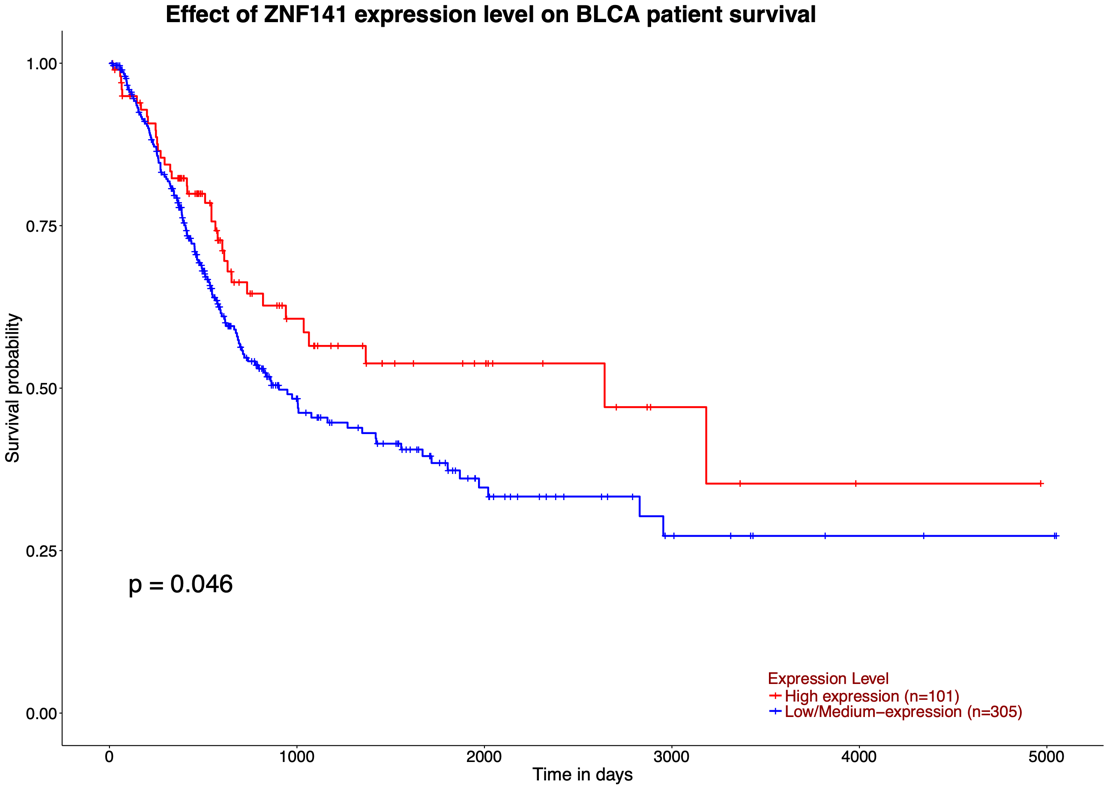


（I）Pheochromocytoma and Paraganglioma (PCPG)


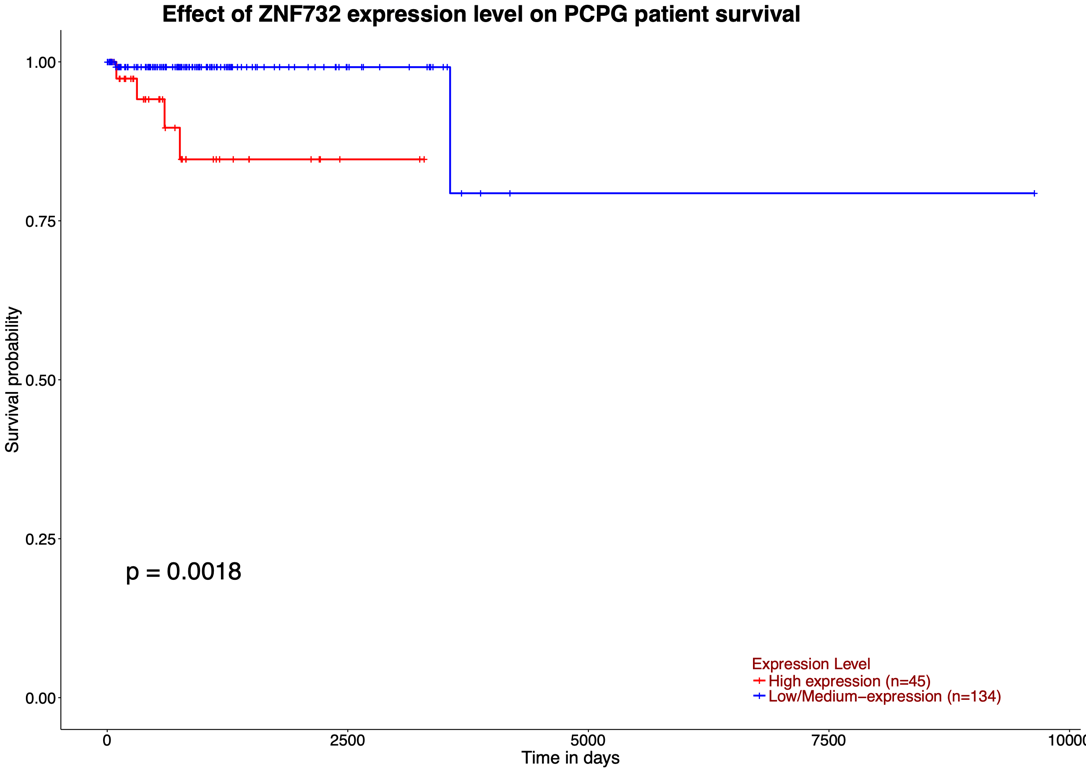


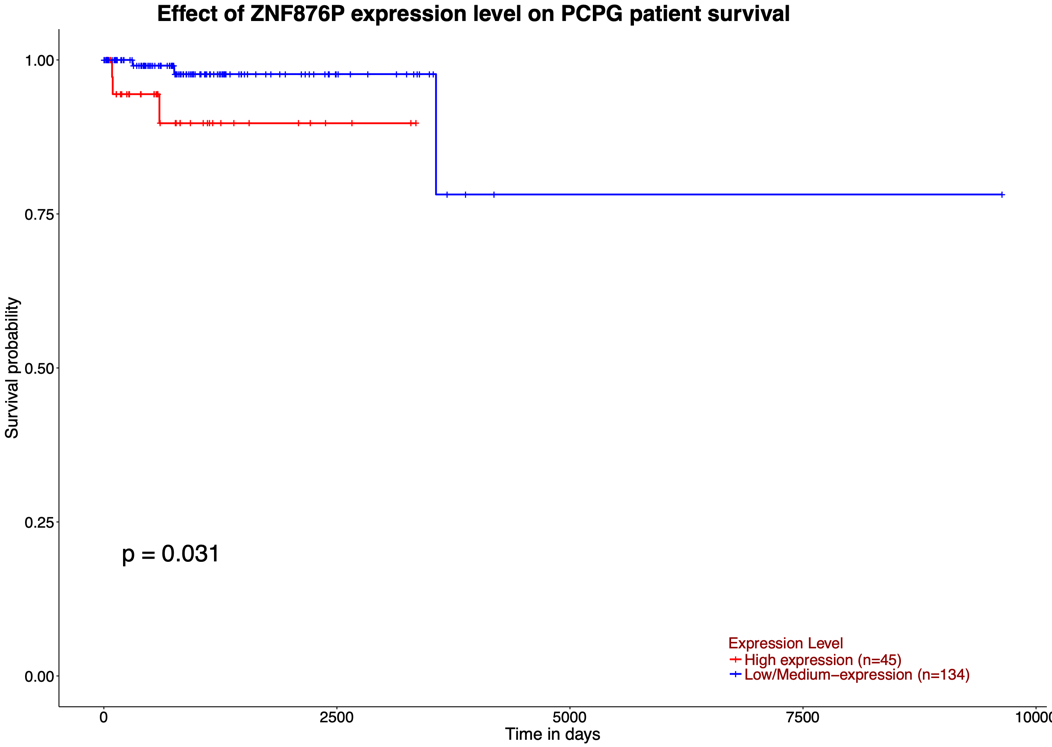


1. Mesothelioma (MESO)


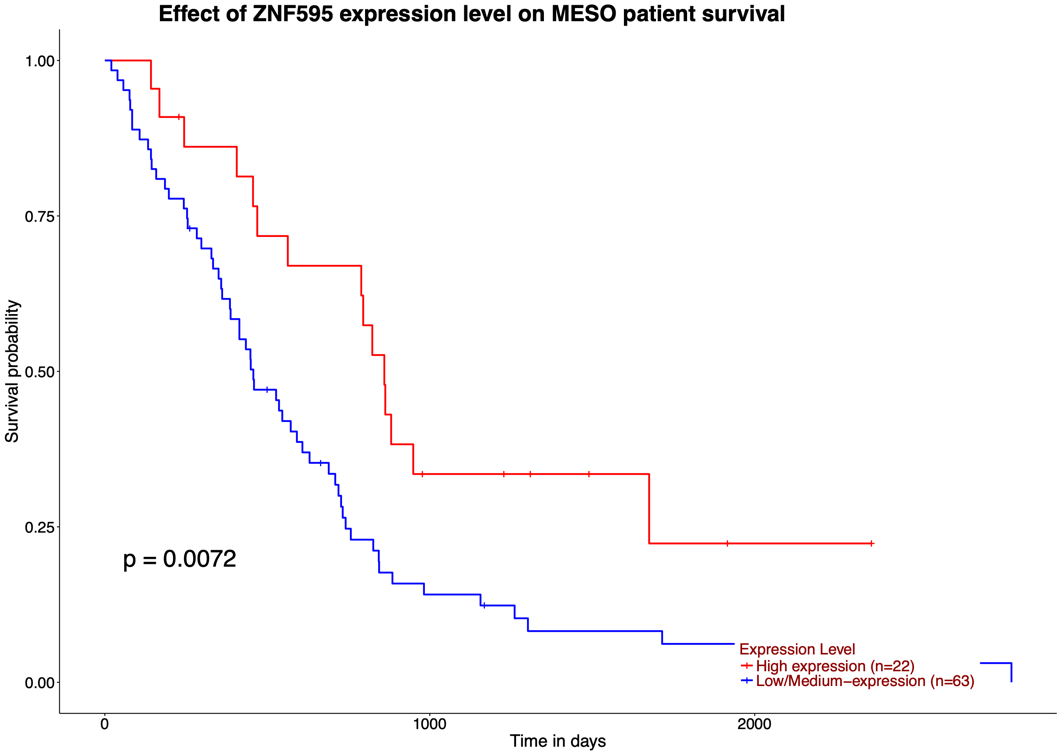


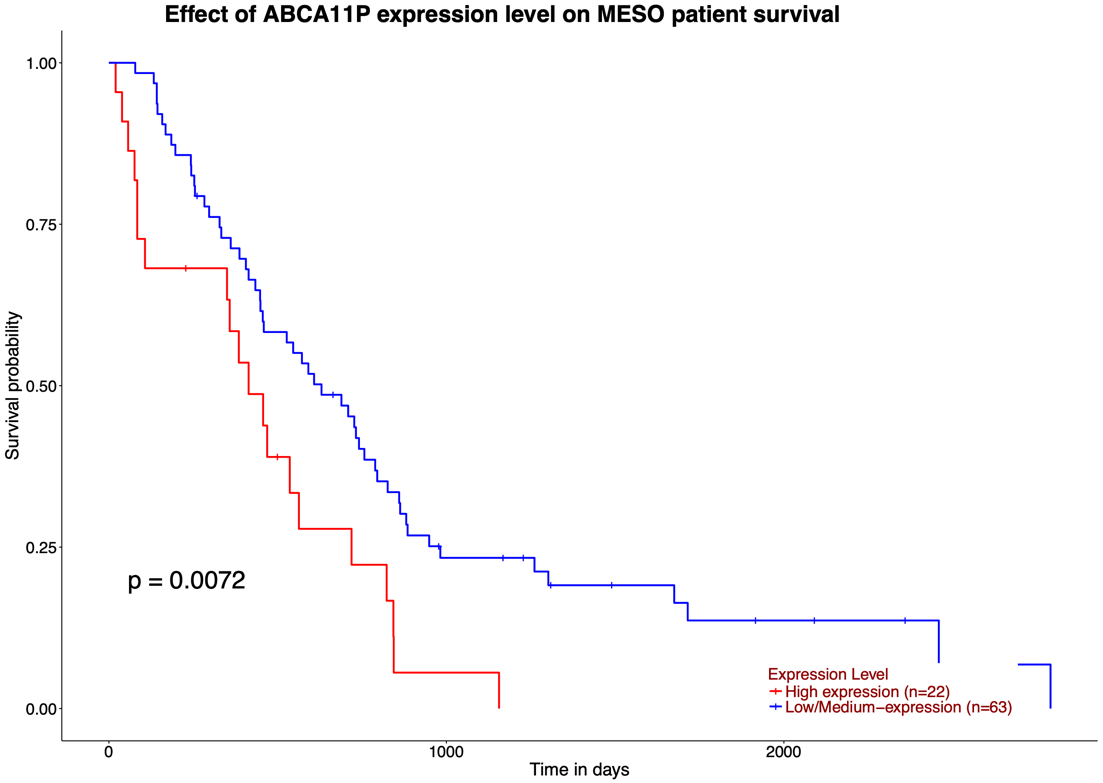


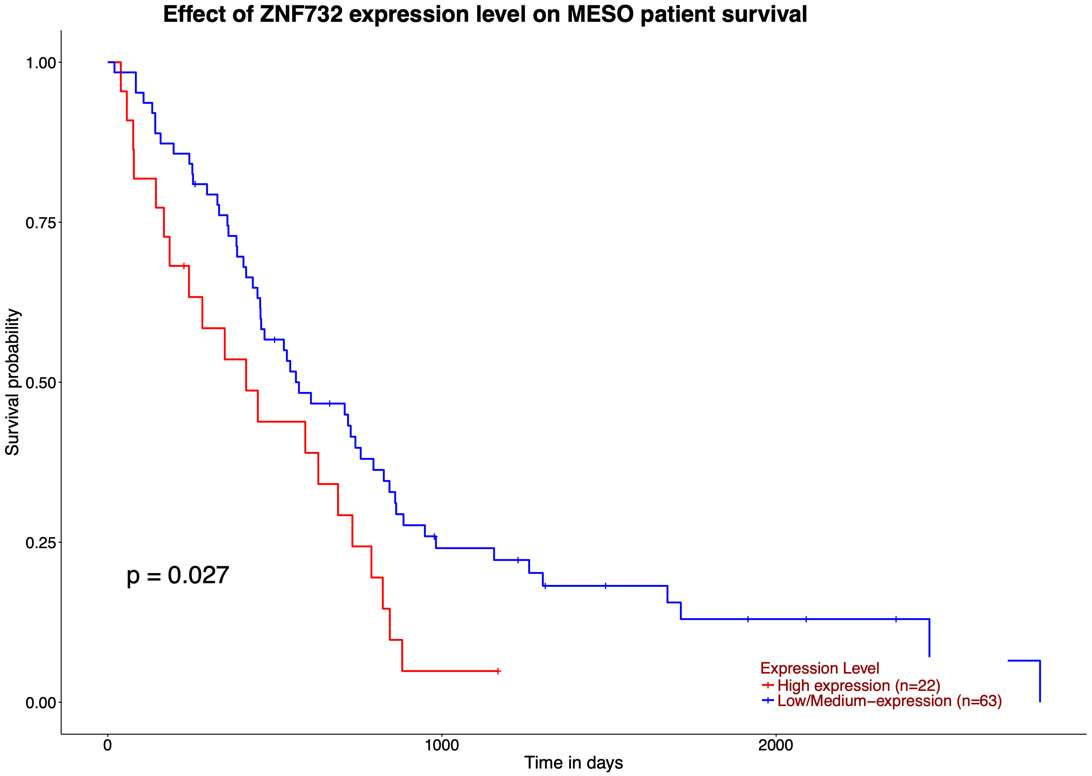


1. Colon adenocarcinoma (COAD)


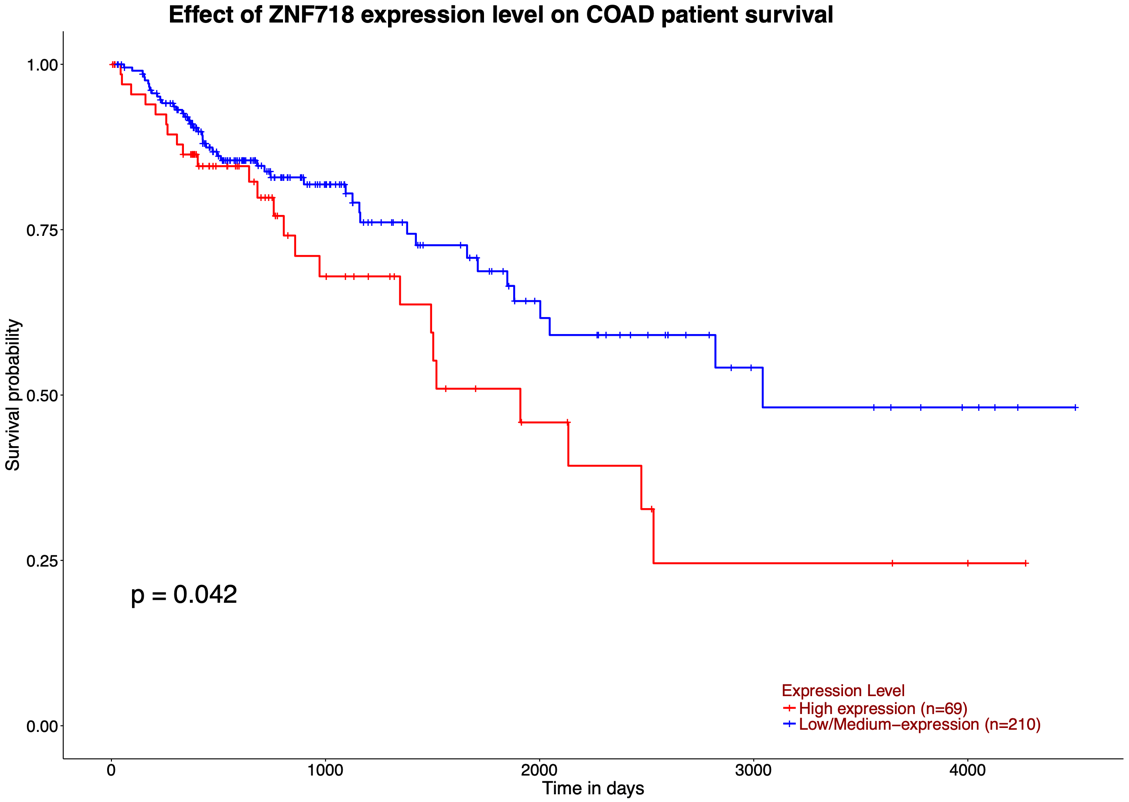


1. Kidney Chromophobe (KICH)


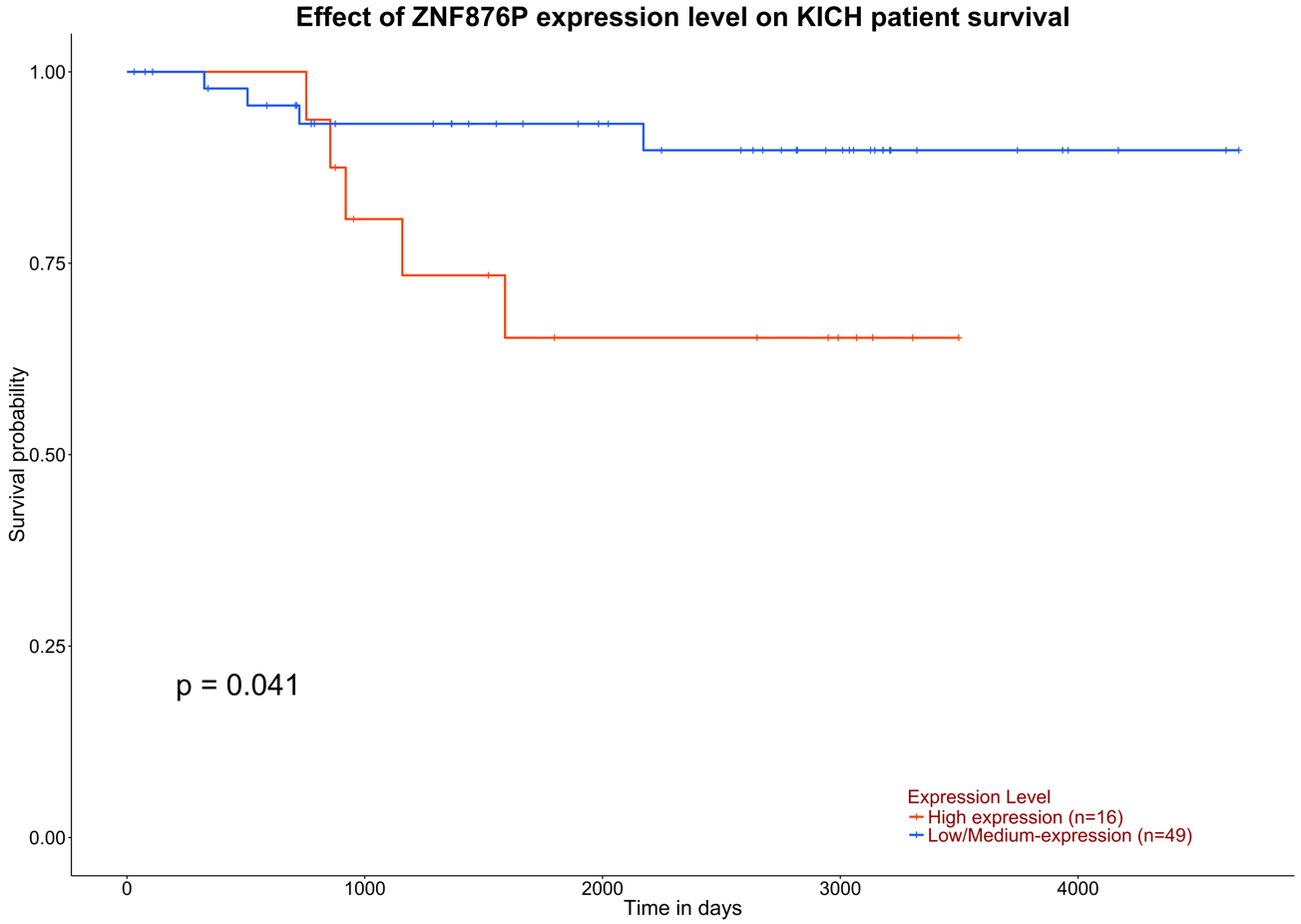


1. Liver hepatocellular carcinoma (LIHC)


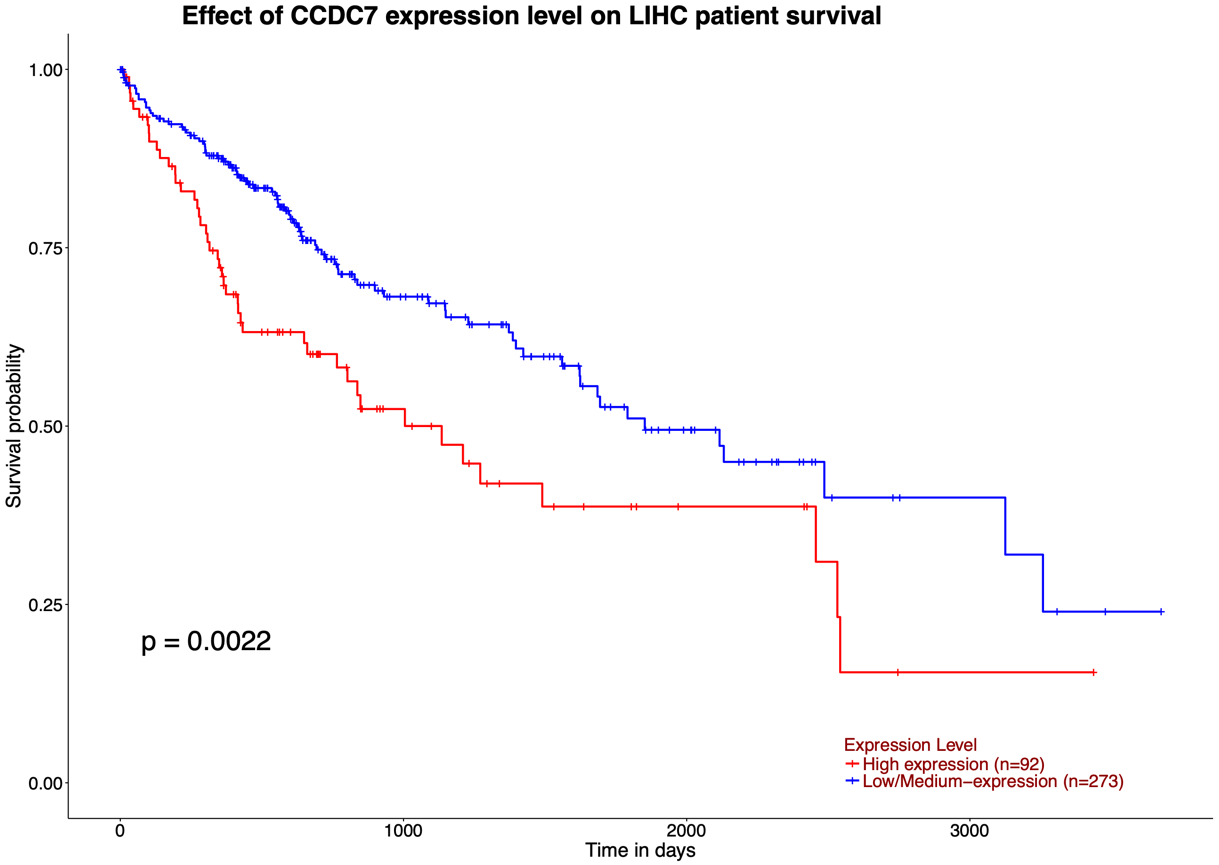

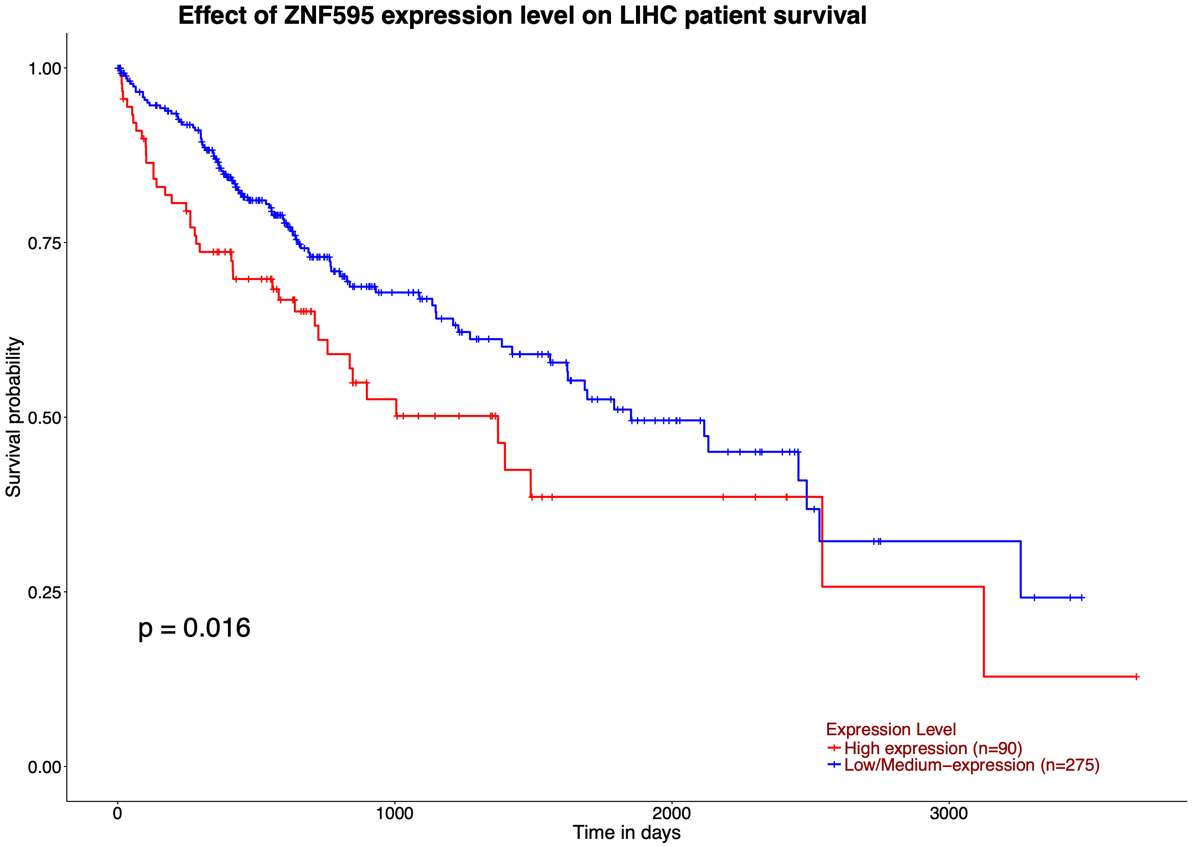


1. Sarcoma (SARC)


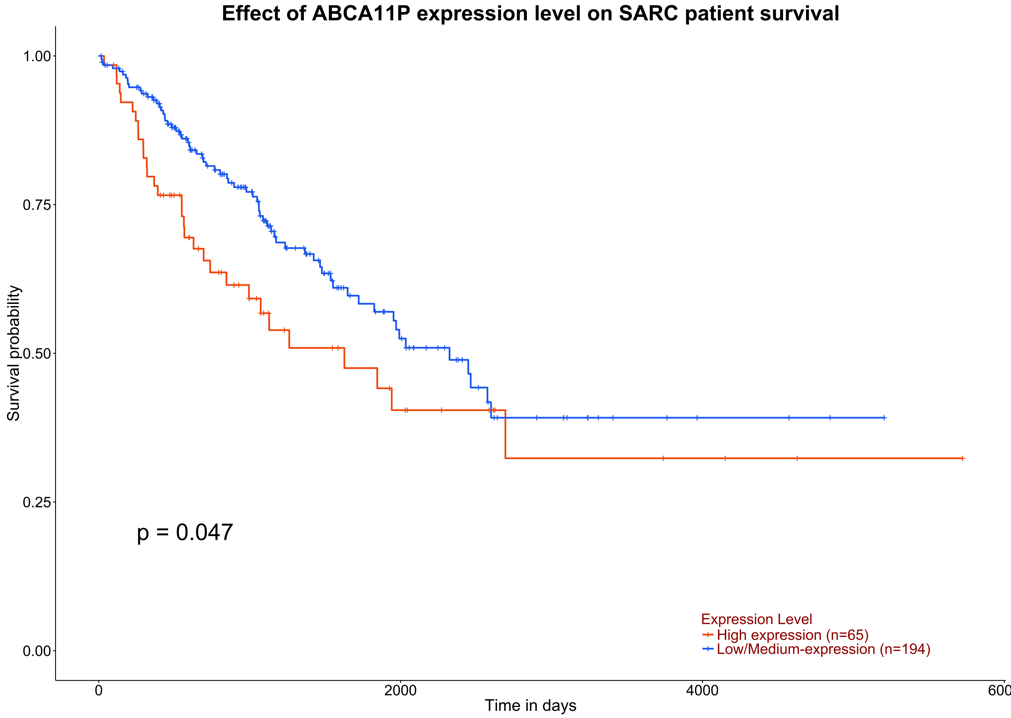


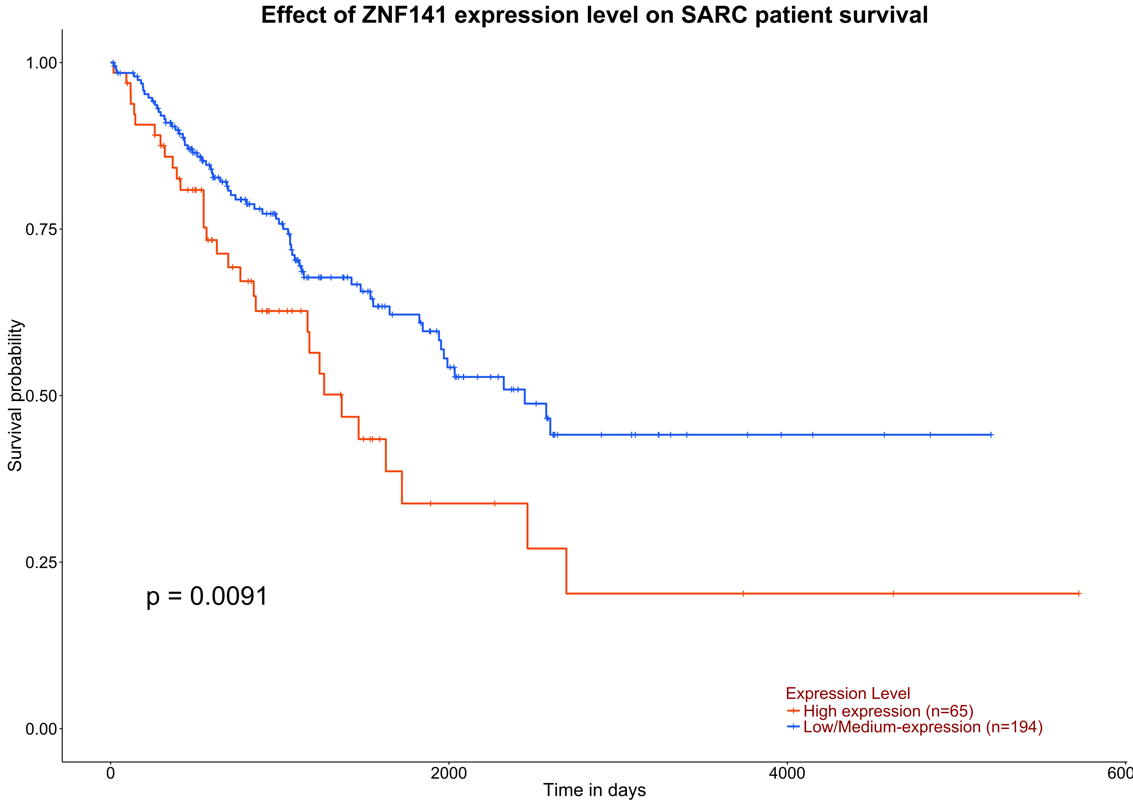


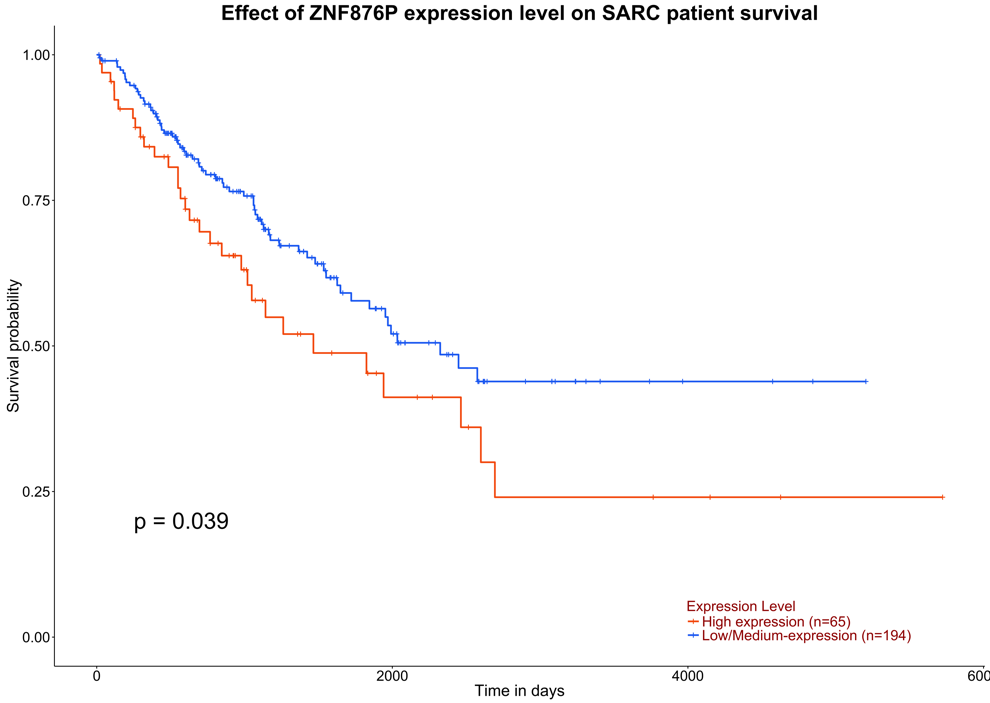


1. Thyroid carcinoma (THCA)


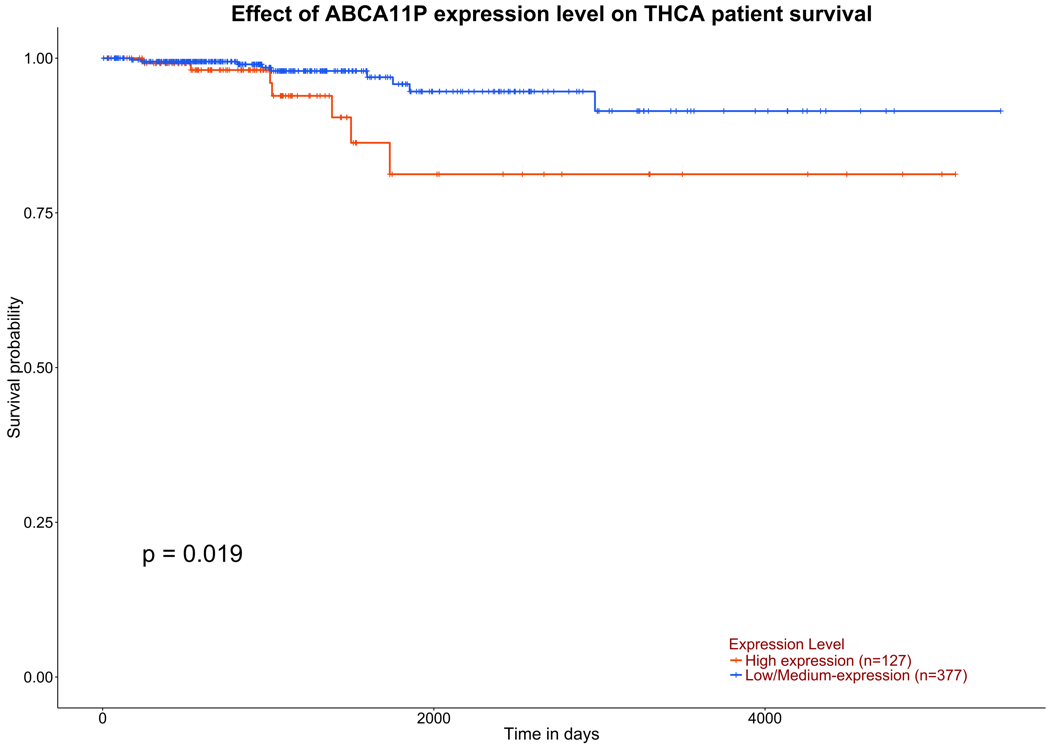


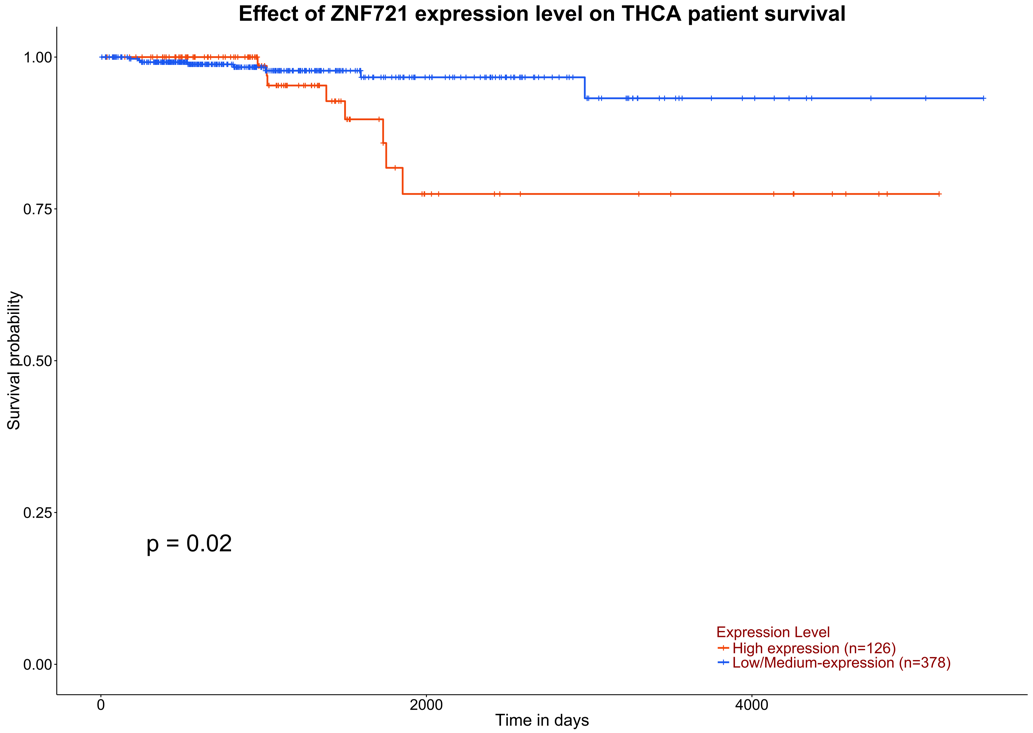

Supplement: Supplementary file 1 [file Data_Sheet_1.DOCX]
